# Applications of deep-learning approaches in horticultural research: a review

**DOI:** 10.1038/s41438-021-00560-9

**Published:** 2021-06-01

**Authors:** Biyun Yang, Yong Xu

**Affiliations:** 1 College of Mechanical and Electronic Engineering, Fujian Agriculture and Forestry University, 350002 Fuzhou, China; 2 Institute of Machine Learning and Intelligent Science, Fujian University of Technology, 33 Xuefu South Road, 350118 Fuzhou, China

**Keywords:** Plant sciences, Plant domestication

## Abstract

Deep learning is known as a promising multifunctional tool for processing images and other big data. By assimilating large amounts of heterogeneous data, deep-learning technology provides reliable prediction results for complex and uncertain phenomena. Recently, it has been increasingly used by horticultural researchers to make sense of the large datasets produced during planting and postharvest processes. In this paper, we provided a brief introduction to deep-learning approaches and reviewed 71 recent research works in which deep-learning technologies were applied in the horticultural domain for variety recognition, yield estimation, quality detection, stress phenotyping detection, growth monitoring, and other tasks. We described in detail the application scenarios reported in the relevant literature, along with the applied models and frameworks, the used data, and the overall performance results. Finally, we discussed the current challenges and future trends of deep learning in horticultural research. The aim of this review is to assist researchers and provide guidance for them to fully understand the strengths and possible weaknesses when applying deep learning in horticultural sectors. We also hope that this review will encourage researchers to explore some significant examples of deep learning in horticultural science and will promote the advancement of intelligent horticulture.

## Introduction

Horticultural crops are an important part of our daily life and mainly include fruits, vegetables, materials for beverages and fragrances, herbal medicine, and ornamental plants. With the progress of modern society, horticultural crops not only play an economic role in providing foods but also play a social role in shaping human culture, beautifying landscapes, and influencing the lifestyles of humans^1,2^. This change in roles, which is becoming increasingly important, has driven horticultural workers to produce more varieties and better products. It also encourages horticultural researchers to do more practical work to improve the functional applications of horticultural crops.

However, in the process of planting horticultural crops, much delicate work needs to be done manually and relies heavily on experienced workers completing jobs, such as pruning branches, thinning flowers and fruit, picking fruit, and preventing insect and pest infestations. Unfortunately, many young people are no longer engaged in gardening; however, with the progress of technology, many advanced and automatic instruments and equipment have been developed and applied to horticulture. To meet the forthcoming demands and challenges, horticultural researchers need to divert their attention towards new technologies to help make better orchard management decisions and revolutionize horticultural productivity. Therefore, producing high-quality fruits, vegetables and ornamental crops by employing advanced technologies, equipment and systems to reduce the use of human force and to improve its efficiency is the primary goal of intelligent horticulture. The rapid, accurate and automatic identification of horticultural crops and the acquisition of planting and postharvest data are important directions of intelligent horticulture^3,4^. Based on new computer technologies and data analysis methods, the development of intelligent systems has provided golden opportunities to improve the cultivation and management of horticulture crops^4^.

The collection of information from orchards or groves has been increasingly facilitated in the past few decades owing to the advancement of various types of sensors in the range of digital horticulture^3^. Modern techniques, including remote sensing, which is implemented by means of unmanned aerial vehicles (UAVs), planes and satellites; the Internet of Things (IoT); thermal and near-infrared cameras; and X-ray imaging technology, have been widely used to collect different kinds of digital information from horticultural crops^3,5^. Based on the collected data, researchers have built models and have applied them to actual production processes. For example, when the growth of horticultural crops is monitored, the growth status of crops can be judged by the established model so that optimal management decisions can be made to optimize the growth process.

However, with the rapid increase in the capability, miniaturization, and sophistication of imaging sensors, a large amount of digital horticultural information has been collected^3,5,6^; therefore, the horticultural science community is facing inundation by a large amount of data, and the data themselves contain various irrelevant and redundant information^6,7^. Thus, creating suitable analytical technologies for such data is extremely important, and the need to deal with and to extract useful features from such uncleaned data is urgent. It is also a practical challenge to convert these technologies into real-world applications.

To date, different kinds of data analysis approaches, including machine-learning approaches, such as partial least squares (PLS)^8^, artificial neural networks (ANNs)^9^, support vector machines (SVMs)^10^, random forests (RFs)^11^, and k-nearest neighbors (KNNs)^1,12–14^, have been developed to tackle the challenges caused by the large amount of heterogeneous data. These approaches have shown great value in processing big data. As a subset of the machine-learning approaches, deep learning has also been widely employed, and has attracted more attention from various domains, such as agricultural production^15–17^, food science^7,18^, robotics^19,20^, and human action and speech recognition^21,22^. As an emerging versatile tool for assimilating large amounts of heterogeneous data, deep learning is able to provide reliable prediction results for complex and uncertain phenomena^6^. In contrast to traditional machine-learning approaches, deep learning contains “deeper” layers in its network structures that provide hierarchical representations for the data by means of various convolutions^16,23^. Deep-learning approaches have shown significant advantages in processing different kinds of big data collected by digital cameras and spectroscopy and have achieved better performance and higher precision than other machine-learning approaches.

Presently, deep learning has already been introduced to horticultural sectors to analyze RGB and spectral images collected from horticultural crops^9,11^. The authors were encouraged to prepare this survey because deep learning has been applied in horticultural science with promising results in ~70 studies. Moreover, since understanding the principles and practical applications of deep learning is not an easy task for researchers and workers in horticultural sectors, many studies are still in development. The aim of this survey is to comprehensively present an overview of the most recent research advances in the application of deep learning to horticultural sciences and provide overall guidance for researchers in this field.

## Brief overview of deep learning

Machine learning is a promising tool for data processing^24^. However, traditional machine-learning methods often require manual feature extraction. With the increase in the amount of large datasets and the advent of graphics processing units (GPUs), algorithmic techniques and methods have been steadily improved. Deep learning was extended from classical machine learning by adding some “deeper” (more complex) structures to models to automatically achieve feature extraction from raw data and has shown better performance than traditional machine learning for some classification and prediction problems^24^. By applying different levels of abstraction layers, various nonlinear functions can be applied to allow data to be represented in a hierarchical way^16,25,26^. This characteristic has proven useful in improving the modeling performance for many large-scale data analysis tasks^27^.

Deep learning is essentially a kind of nonlinear information processing technique based on representation learning and pattern analysis. Typically, deep-learning models are built to refine multilevel representations with multilayer neural networks. Neural networks are generally composed of multiple neurons arranged in layers. Two adjacent layers are connected by neurons in terms of weights that need to be trained to learn maps, which are usually complex, from inputs, which are generally pre-extracted and processed data or features, to outputs or labels. Neurons that essentially represent various nonlinear functions and transformations are used to build complex models. By connecting more layers of neurons to form more complicated models that allow massive parallel connections, deep learning can solve complex real-world problems in a rapid and effective way^16,28^.

The property of a highly hierarchical structure along with the massive learning capability of deep-learning models enables them to carry out predictions and classifications particularly well with good flexibility and adaptability to a wide range of highly complicated data analysis tasks^28^. With the robust capability of automatic feature learning, many complex problems in the field of horticultural science can be solved in an effective and rapid way by utilizing deep-learning methods, including various recognition^29–31^, yield estimation^32,33^, quality detection^27,34^, stress phenotyping detection^35,36^, growth monitoring^37,38^, and other applications^39,40^. In the next section, we introduce these applications in detail.

Convolutional neural networks (CNNs) and their derived models are considered key deep-learning approaches in the field of artificial intelligence and have led to breakthroughs in image processing and analysis. CNNs are a family of multilayered neural networks constituting a class of deep, feed-forward artificial neural networks (ANNs) that have been successfully applied to computer vision applications^5,25,26^. CNNs are currently recognized as one of the most significant machine-learning approaches for big data analysis in a large variety of research areas^28^. Of our surveyed papers, the application of CNNs and their derivatives in horticulture accounts for a large proportion (65 papers, 92.86%). CNNs typically contain a number of common components, including convolution, pooling and fully connected layers, in different configurations that are connected successively to perform some complex-learning tasks.

**Fig. 1:**
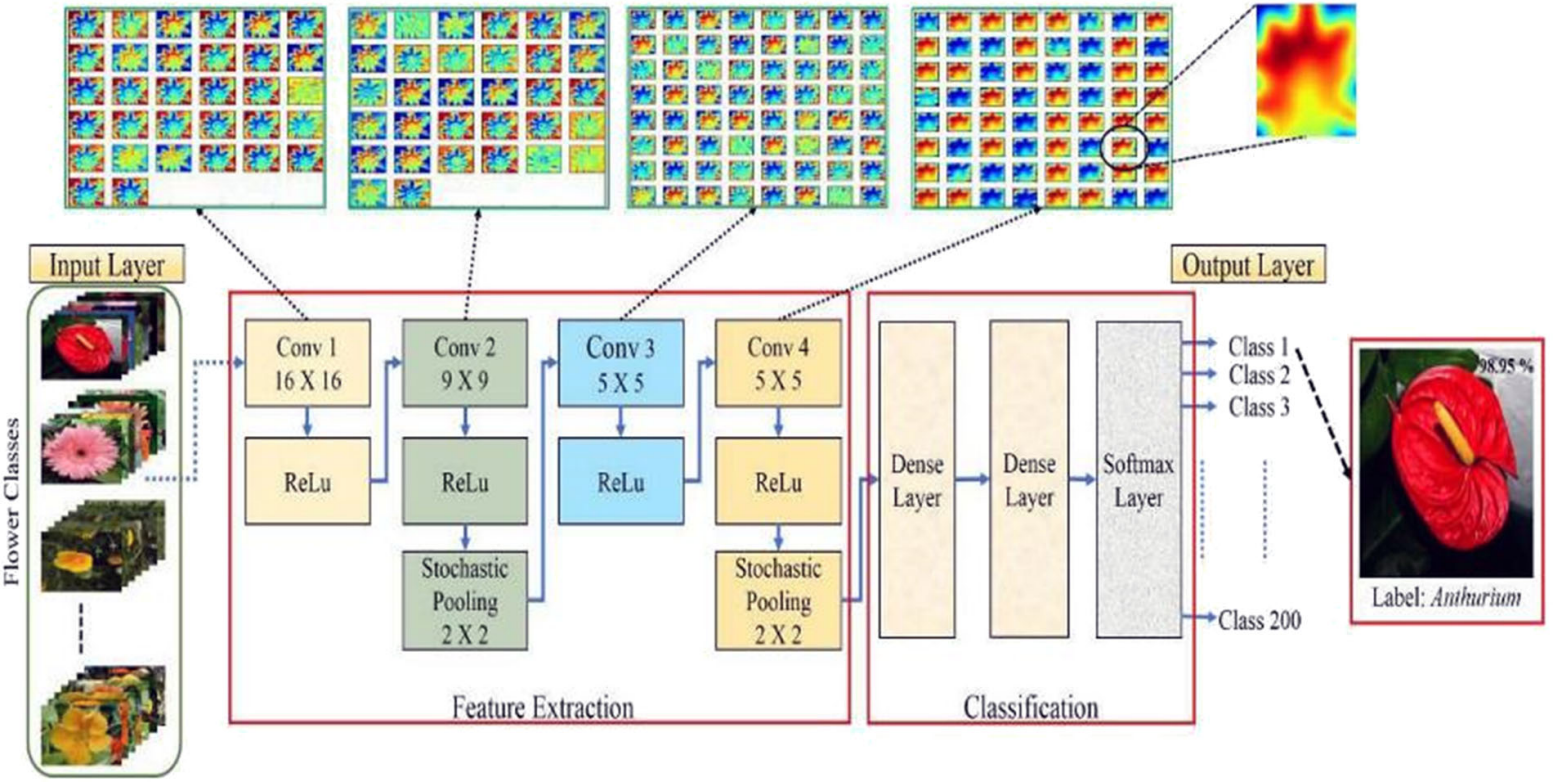
A DCNN architecture. The model contains one input layer, four convolutional layers, four ReLU components, two stochastic pooling layers, two fully connected layers and one softmax regression output layer. Source: ref. ^29^

A typical deep CNN (DCNN) architecture is shown in Fig. 1. To correctly classify different species of flower images, by acquiring previous knowledge from LeCun et al.^24^, Prasad et al.^29^ proposed a multistage CNN architecture composed of one input layer, four convolutional layers with various window sizes, five rectified linear unit (ReLU) components, two stochastic pooling layers, one densely connected layer and one layer of softmax regression output^29^. The input of a typical CNN is generally two-dimensional image data. The convolution layers are the core of a whole CNN and are composed of 2-dimensional kernels with varied weights that are moved over the image and perform feature extraction. In Fig. 1, the sizes of the four convolution kernels are set to 16*16, 9*9, 5*5, and 5*5. After convolution, a pooling layer may be used to compress the amount of information by reducing the dimensionality of the inputs to avoid overfitting. This process is achieved by substituting multiple neurons within a subsampling window with a single output neuron. In Fig. 1, 2*2 stochastic pooling is used in which every 2*2 neuron in the original layer is substituted by only one random value taken from the neuron in the new layer, which reduces the number of neurons in the new layer by a factor of 4 and makes the calculation converge quickly. Finally, these features are fed to some fully connected layers for classification.

**Fig. 2:**
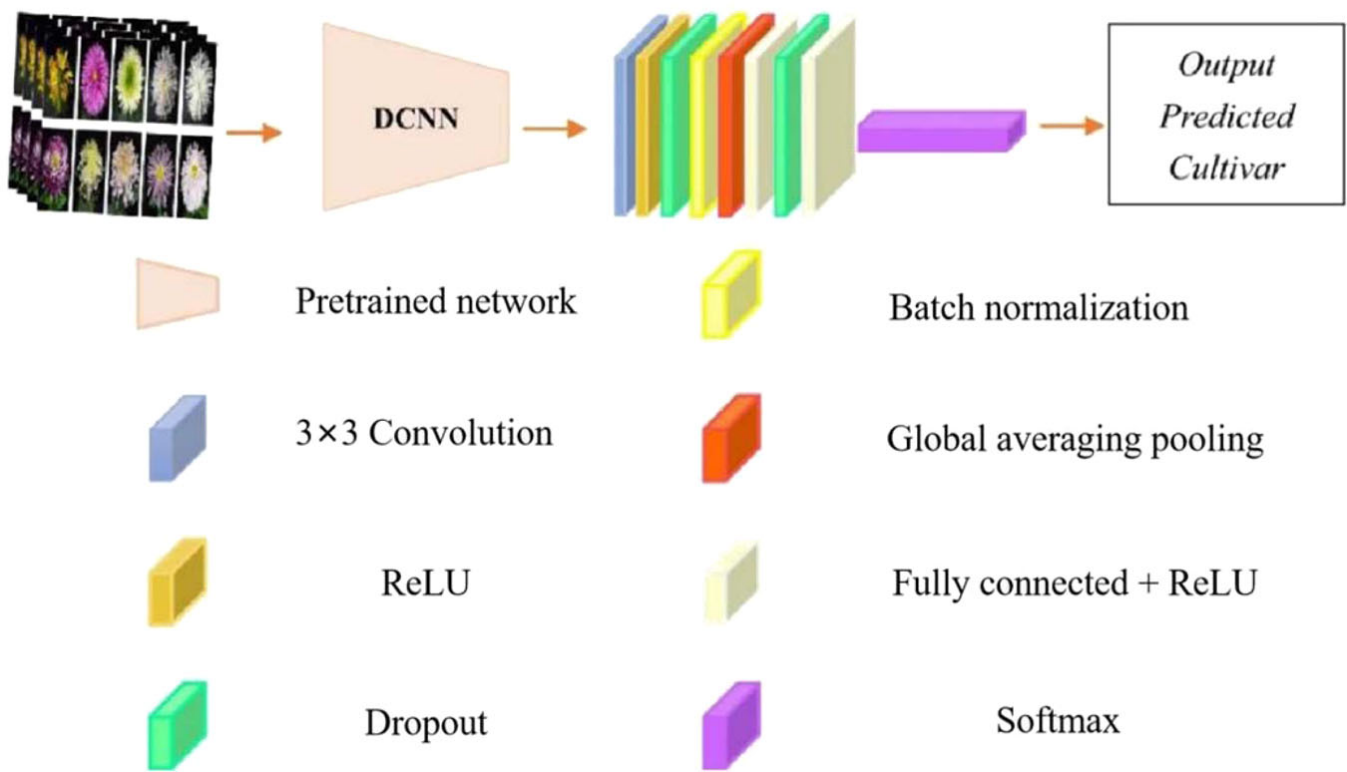
A DCNN framework. The performance and stability are improved by the batch normalization layer. Overfitting is prevented by the dropout layer. Global average pooling can adapt to different input image sizes. Source: ref. ^31^

Generally, the choice of hyperparameters in training deep-learning models to a large extent determines the performance of the trained model. Important hyperparameters include the network architecture (such as the numbers of neurons in hidden layers, the structures of convolution and pooling layers and the number of layers), and contain the learning rate, weight initialization and activation function^17^. Although self-established architectures may be innovative and groundbreaking, they usually require a high level of computer literacy that is difficult for normal horticultural researchers to use. Thus, researchers often begin with a pretrained architecture that has been shown to perform well across a large variety of data structures and problems and adapt it to the problem considered, which has been demonstrated as a reliable and feasible common practice^17,31^. As Fig. 2 shows, feature extractors were created by the pretrained network for chrysanthemum recognition^31^, the classifier consisted of two fully connected layers (each having 4096 hidden neurons), batch normalization units were used to increase DCNN performance and stability, a global averaging pooling layer was adapted to images with different input sizes, and a dropout layer was used to prevent the CNN model from overfitting.

**Fig. 3:**
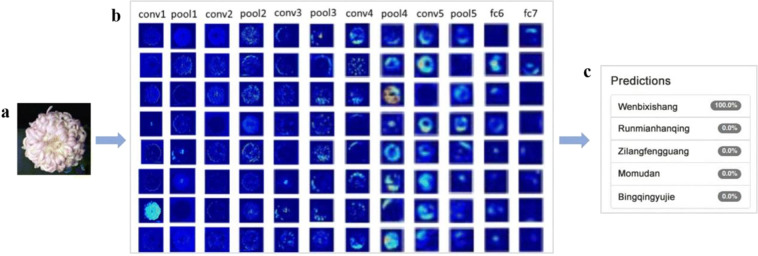
VGG-16 model for image recognition. **a** The input images. **b** Visualization of the feature extraction results after each convolution (conv), pooling (pool) or fully connected (fc) layer. **c** The top-k prediction results. Source: ref. ^31^

Examples of CNN architectures that have been used for classification or regression tasks include LeNet^36,41^, AlexNet^30,42^, VGGNet^43–45^, GoogLeNet^46^, and ResNet^47,48^ (the typical CNNs and corresponding parameters are shown in Table 1). For instance, Fig. 3 shows visual examples of flower images after each step of the Visual Geometry Group Network 16 (VGG-16) process for chrysanthemum cultivar recognition^31^. The network was composed of five convolutional layers, each of which was followed by one pooling layer. The convolution layers were used as filters to extract features from the input images, and the output from each convolution layer was downsampled by a pooling layer for dimensionality reduction. After the processes of five successively connected convolution and pooling layers were performed, two fully connected layers were used as a classifier to exploit the learned highly abstract features to classify the input images into predefined categories or to conduct some numerical predictions^7,16^. The parameters (the number of channels, activation functions, kernel size, padding strides, etc.) inside the convolution layers and the selection of the applicable models should be optimized according to the particular problem^7^. For example, when the VGG network was created, it was the deepest network available and showed competitive performance with only 3*3 stacked convolution kernels, which is better than using a large convolution kernel. In addition, the residual network (ResNet) incorporated local residual connections that not only improve its learning speed but also allow the network to become significantly deeper for extracting more high-level features, which enable ResNet to have a higher predictive power.

**Table 1:** Typical CNNs and their corresponding parameters

No.	Network name	Meaning	Year	Network layers	Convolutional layers	Top-5 rate on ImageNet	Feature	Source code	References
1	LeNet	The Network proposed by Yann LeCun et al.	1998	7	3	NA	•The first CNN •The activation function is sigmoid	https://github.com/omarsemma/Lenet5	^110^
2	AlexNet	The Network proposed by Alex	2012	8	5	83.6%	•Nonlinear activation function: ReLU •Prevent overfitting: Dropout •Big data training: ImageNet	https://github.com/mosbys/AlexNet	^111^
3	VGGNet	Visual Geometry Group Network	2015	19	16	92.7%	•All use a 3*3 convolution kernel and a 2*2 pooling kernel •Deeper network structure •The depth layers range from 11 to 19	https://github.com/wanglimin/Places205-VGGNet (https://arxiv.org/abs/1508.01667)	^112^
4	GoogLeNet	The deep network structure developed by Google	2015	22	21	93.3%	•Adopts a modular structure (inception structure) •Uses average pooling instead of a fully connected layer	https://github.com/kevin28520/GoogleNet (https://arxiv.org/abs/1409.4842)	^113^
5	ResNet	Residual Network	2015	152	151	96.43%	•Adds a residual network in the model •Makes a reference to the input of each layer to form a residual function •Uses the shortcut connection method	https://github.com/KaimingHe/resnet-1k-layers (https://arxiv.org/abs/1512.03385)	^114^

**Fig. 4:**
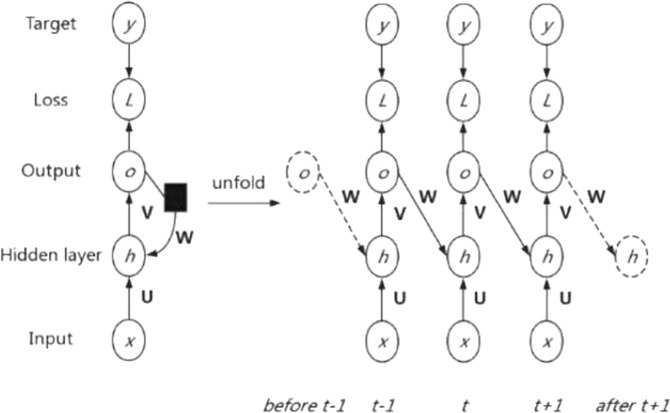
Structure of an RNN. The information of the RNN propagates upwards from the initial input state. The only feedback of data is from the output neurons to the hidden neurons. The activation functions for the hidden and output neurons are the hyperbolic tangent and pure linear functions, respectively. Source: ref. ^39^

Another deep-learning model widely used for processing sequential or time-series data is the recurrent neural network (RNN)^39,49^, which has been extensively applied in price prediction^49^, natural language processing^50^, speech recognition^51^, and other fields^52^. The basic principle behind an RNN is that previous information can be memorized by a network and utilized to calculate the current output. To do this, the input of the hidden layer comprises both the output from the hidden layer at the last moment, which represents the memorized previous information, and the output of the current input layer^39^. The tanh activation function is normally adapted in hidden neurons, while for output neurons, the activation function is generally selected according to the problem to be solved^49^. The feedback from the output neurons to the hidden neurons is the only loop in the RNN. The diagram of the RNN structure used for forecasting horticultural product prices is displayed in Fig. 4.

**Fig. 5:**
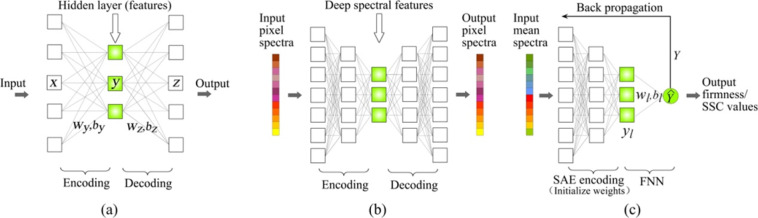
The SAE-FNN architecture. **a** The autoencoder structure, **b** SAE is pretrained in an unsupervised manner with random pixel spectra, **c** SAE-FNN is fine-tuned in a supervised manner with mean spectra and firmness (or SSC). Source: ref. ^27^

Figure 5 shows another deep-learning architecture named SAE-FNN, which is composed of a stacked autoencoder (SAE)^53^ and a fully connected feed-forward neural network (FNN). The network was used to predict the soluble solid content (SSC) and the firmness of a pear^27^. SAE uses an unsupervised approach with a basic structure consisting of an autoencoder, which is used for extracting features from the input data by the nonlinear processing of deep neural networks^27,53^. As Fig. 5a shows, the input was encoded into a lower dimensional vector, which was then extended again by a decoder to reconstruct the original input. Therefore, the vector, in which the decoding part is removed and the encoding part is retained, is used as the extracted feature of the input. The extracted features output from the trained network are fed to the FNN to form an SAE-FNN network for prediction tasks (shown in Fig. 5c).

**Fig. 6:**
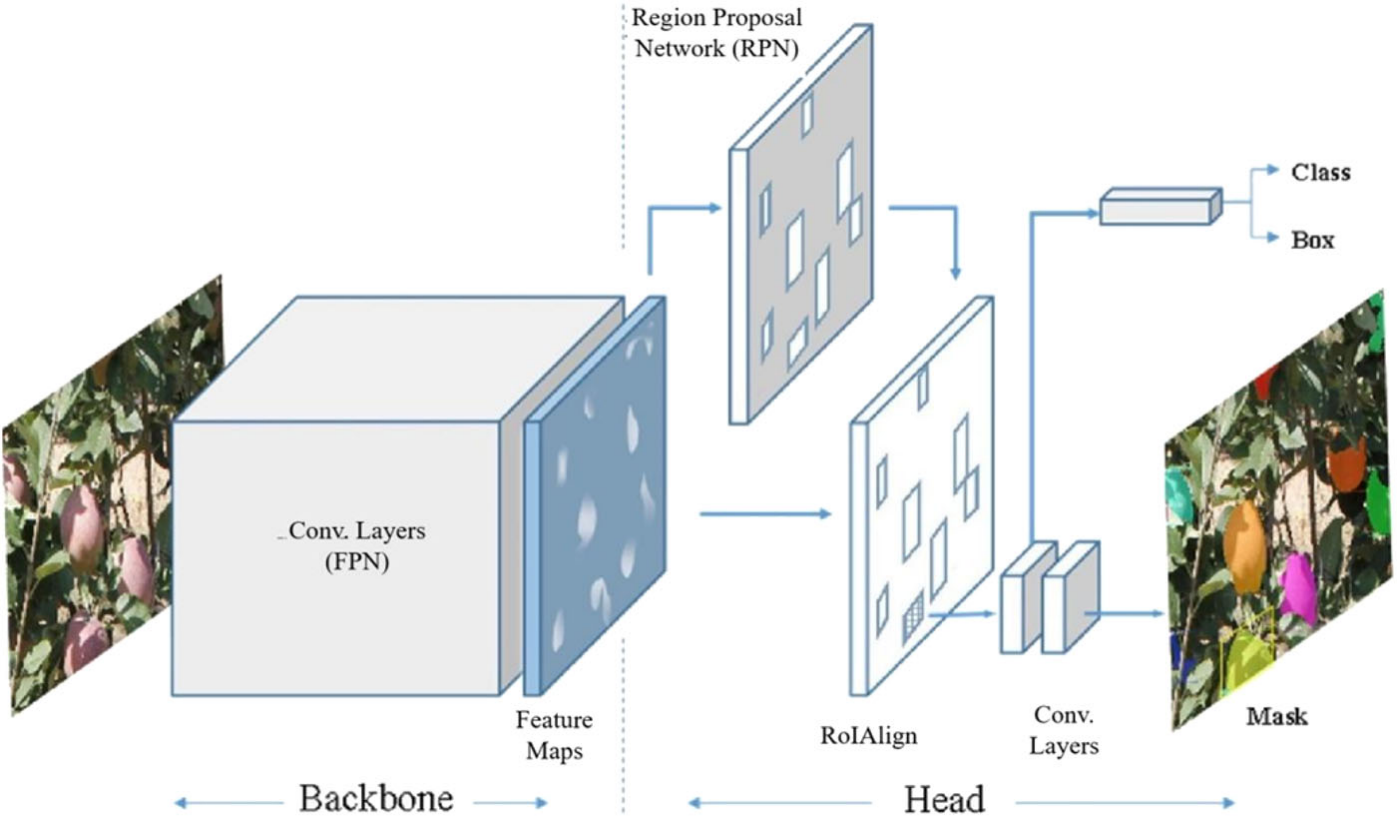
The Mask R-CNN architecture. The architecture consists of two parts: the backbone and the network head. Source: ref. ^68^

In addition to being used for classification and regression tasks, deep-learning techniques have also been used for image segmentation tasks. The R-CNN method is a two-stage deep-learning object detection method (a computer vision technique for locating instances of objects in images or videos) that combines a CNN with the region proposal method. At present, the Mask R-CNN (mask region-based CNN) is the state-of-the-art R-CNN method in the field of image segmentation. By adding a branch, Mask R-CNN extends Faster R-CNN in order to exactly generate a high-quality segmentation mask on each region of interest (RoI)^54,55^. Figure 6 shows the architecture of the Mask R-CNN, which consists of two parts: the backbone (a feature pyramid network (FPN), which is a fully convolutional network) for feature extraction and the network head (a small network sliding over the feature map) for bounding-box (a compact rectangular box that contains the object) recognition and mask prediction. In this figure, the Mask R-CNN model can detect and segment fruit automatically, and the mask images of the fruit are output from the model with bounding boxes. Moreover, Mask R-CNN can extract object regions from the background at the pixel level.

From the brief introduction of several commonly used models described above, we know that deep-learning technology has a powerful role in image classification, regression and segmentation. Furthermore, there are also many other kinds of network structures that are applied, such as single shot multibox detection (SSD)^56^, long short-term memory (LSTM)^32^, you only look once (YOLO, source code is available at: http://pjreddie.com/yolo/)^57^, regions-CNN (R-CNN)^58^, fast region-based CNNs (Fast R-CNN, source code is available at: https://arxiv.org/abs/1504.08083)^33^, faster region-based CNNs (Faster-RCNN, source code is available at: https://github.com/shaoqingren/faster_rcnn (in MATLAB) and at https://github.com/rbgirshick/py-faster-rcnn (in Python))^44,59,60^, and so on. In addition, the processed data types are not simply limited to RGB images but can also include any other data forms, such as video, hyperspectral images, and spectral data.

Finally, it is helpful to introduce and describe some of the evaluation metrics used to examine network performance. Some performance metrics that were used in our reviewed papers are defined as follows:

- Classification accuracy (CA): This is a measure of the number of correctly classified images/classes over the total number of images/classes for classification results. For multiclass classification problems, CA is averaged over all the classes.
- Precision: The fraction of true positives (TP) over the sum of the TP and false positives (FP). *P* = TP/(TP + FP).
- Recall: The fraction of TPs over the sum of the TPs and false negatives (FN). *R* = TP/(TP + FN).
- F1-score (F1): The harmonic mean (the weighted average) of precision and recall. The F1-score varies between 0 (worst) and 1 (best). F1 = 2 * (TP * FP)/(TP + FP).
- Root-mean square error (RMSE): Root-mean square of the differences between the predicted values and observed values.

## Applications of deep learning in horticulture crops

Deep-learning technologies have been successfully applied in the horticultural domain with promising results. The application fields of deep-learning approaches in horticultural sectors include variety recognition, yield estimation, quality detection, stress phenotyping detection, growth monitoring and others. In this section of the paper, we review the specific problems addressed in the literature, the architectures and models implemented, the sources of data employed, the overall performance achieved on the basis of the metrics adopted, the comparisons with other methods, and the links to or sources for the original code for some examples. However, it is difficult to compare different studies because the datasets and performance metrics used in different studies generally vary. Thus, in this section of the paper, we focus only on comparisons of techniques applied on the same dataset within the same research paper so that the same metrics are used. Some other evaluations and comparisons among different technologies from different papers can be found in “Summary and discussions” subsection.

### Recognition and classification of horticultural crops

Automatic recognition and classification of horticultural crops according to different features are the major challenges in horticultural research. The recognition of horticultural crops is a challenging task due to the great variety of crop types. There are at least 91 species of fruit-bearing plants, >200 vegetable plants, and >6000 ornamental cultivars, and many cultivars are created and disappear each year^2^. Moreover, horticultural crops can mutate in many ways, resulting in a large amount of intraclass variation. For example, similar features can be shared within flower classes, i.e., different species of flowers can share similar shapes, colors and appearances. Therefore, classifying horticultural crops is a multiclassification problem. Even though it is feasible to conduct manual classification, it is generally very time consuming and error prone when dealing with a large number of samples^43^. Therefore, the application of deep-learning methods to species or cultivar recognition and classification will be an unprecedented breakthrough in horticultural research due to their high speed and robust recognition performance^31^.

Currently, due to the successful application of CNNs, the accuracy of image classification and event prediction has been greatly improved. With ImageNet, which is an open data resource (http://www.image-net.org/), five categories of vegetables, including broccoli, pumpkin, cauliflower, mushrooms and cucumber^30^, were used to train a DCNN (the AlexNet model). The experimental results showed that the accuracy rate of this DCNN model on the vegetable image dataset reached 92.1%, which was a significant improvement compared with the SVM classifier (80.5%) and the back propagation (BP) neural network (78%) methods. Prasad et al.^29^ utilized 9500 images from the KLUFD and OUFD flower datasets to train a CNN model with a system architecture of four convolution layers with different filtering window sizes and employed a stochastic pooling technique for classification (see Fig. 1). The average flower recognition accuracy rate was 97.78% for the proposed CNN model, which is higher than those of other advanced classifiers. For flower species classification, Cıbuk et al.^61^ employed the concatenated AlexNet and VGG-16 models to extract features, which were then used as the input to the minimum redundancy maximum relevance (mRMR) method for selecting some higher abstract features. The selected abstract features were fed into the SVM classifier that was combined with a radial base function (RBF) kernel to obtain the final classification results. They attained a 96.39% accuracy value on Flower17, and the average accuracy was 95.70% on Flower102. Hiary et al.^43^ built an excellent two-step deep-learning-based model also aimed at flower type identification. The robust classifier contained two steps: an FCN (fully convolutional network) method, which was initialized by the VGG-16 model to segment the flower regions, and a CNN method, which was initialized by segmenting the FCN to classify the flower classes. Compared with other approaches, the proposed model achieved better learning performance and simplified the flower classification task, with classification accuracies of 98.5%, 97.1%, and 99.0% on Oxford 17, Oxford 102, and Zou–Nagy, respectively.

At present, several open-access image datasets with different kinds of horticultural crops, such as ImageNet and OUFD, have been collected by researchers. These large-scale image datasets are widely used and provide plenty of feature information for training deep neural network models for horticultural crop recognition. However, since horticultural crop recognition systems are still in the development stage and have not been established on a large scale, most researchers prefer to experiment with their own collected image sets. Liu et al.^31^ proposed a deep-learning model with a VGG-16 network to recognize large-flowered chrysanthemums from two datasets collected by their own group. Dataset A comprised 14,000 images collected from 103 cultivars by an automatic image acquisition device in 2017 and was utilized to train a model and to determine the model’s calibration accuracy (the top-5 rate, which is the fraction of test images for which the correct label is within the five labels considered most likely by the model, was over 98%). Dataset B contained 197 images of the same cultivars as in dataset A and were obtained with a digital camera in 2008–2010 and 2016; the images were imported into the established classifier to test the model’s generalization ability (the top-5 rate is above 78%). In other applications, different models for classifying three different plum^62^ varieties (*Angelino*, *BlackSplendor* and *Owent*), six different grape^63^ varieties (*Tinta Roriz, Tinta Amarela*, *Tinta Barroca*, *Tinto Cão*, *Touriga Nacional* and *Touriga Franca*) and seven different olive^64^ varieties (*Arbequina*, *Changlot Real*, *Arbosana*, *Picual*, *Lechín de Sevilla, Ocal* and *Verdial de Huévar*) were proposed and used DCNNs. The experimental results showed that the proposed system achieved remarkable behavior, with an accuracy rate ranging from 91 to 97% in plum variety classification. The highest accuracy was 95.91% when applying the Inception-ResNet-V2 architecture to classify olive varieties from 2800 fruits, and the network modified from AlexNet for grape variety identification achieved a test accuracy of 77.30%. In addition to using RGB images, Fernandes et al.^65^ used spectral information to separate the two main grapevine varieties (Touriga Franca (TF_var_) and Touriga Nacional (TN_var_)). Datasets with a total number of 35,833 spectra from 64 varieties and 626 plants were collected to establish the classification model. The results showed that each model had its own merits: for TN_var_, the SVM model achieved better experimental results than the CNN, and 81.9% of the TN_var_ spectra and 63.0% of the non-TN_var_ spectra were correctly classified. For TF_var_, the CNN achieved the best results, and the correct classification percentages of the TF_var_ and the non-TF_var_ spectra were 93.8% and 91.6%, respectively.

In addition to variety identification, deep learning has also been widely used in the automatic detection of orchard fruits. The development of a reliable and accurate fruit detection system is the first step in creating an autonomous harvesting system that is a promising prospect of future agricultural applications. To develop efficient and robust fruit detection systems for orchards, many researchers have performed related studies to address some complex conditions, such as illumination variation, leaf shading, and changing appearance^47,66^, in working environments.

To conduct robust and efficient detection and segmentation of fruits and branches in apple orchards, Kang and his team proposed a series of neural network frameworks based on deep learning, such as LedNet (a network that utilized a FPN combined with atrous spatial pyramid pooling (ASPP) to improve the model’s detection performance) with ResNet-101^67^ (a lightweight backbone), the DaSNet model (a network, which adopted a gated feature pyramid network (GFPN) combined with ASPP to enhance the feature extraction capability of the model) with ResNet-101^66^, the DaSNet model with ResNet-50^66^, the DaSNet-V2 model with ResNet-101^47^, and the DaSNet-V2 model with ResNet-18^47^ (source code: https://github.com/tensorflow/models/tree/master/research/slim)^66^. The experimental results showed that LedNet with ResNet-101 achieved an accuracy of 86.4%, an F1 score of 0.849 and a recall score of 84.1% for the detection of apples in orchards, and DaSNet with ResNet-101 reached 77.2% and 87.6% for the semantic segmentation of branches and apples^67^.

Semantic segmentation in computer vision is the segmentation of different objects at the pixel level where each pixel is uniquely assigned to one object category of the image. In an instance segmentation task, all pixels associated with every object in the image should be distinguished and annotated. Instance segmentation on each fruit is necessary because it can provide abundant geometric property information (such as size and shape), and such parameters can be utilized to identify the poses of the fruits, especially for overlapped or shaded fruits. Then, they proposed an improved deep neural network called DaSNet-V2^47^. The DaSNet-V2 model with ResNet-101 obtained a recall score of 86.8%, a precision score of 88%, a branch segmentation accuracy of 79.4%, and an apple segmentation accuracy of 87.3%. DaSNet-V2 can efficiently and robustly perform visual sensing for automatic harvesting in apple orchards. Furthermore, Gené-Mola et al.^68^ performed a study on apple 2D detection with Mask R-CNN and 3D location utilizing structure-from-motion (SfM) photogrammetry. By testing 11 normally grown Fuji apple trees comprising a total number of 1455 apples, the system achieved encouraging performance with an F1-score that increased from 0.816 for 2D detection to 0.881 for 3D detection and location.

Since fruits and vegetables have different shapes and colors, some scientists have conducted further experiments with specific horticultural products and tried to extend models to the identification and classification of other products.

Mao et al.^69^ proposed an automatic cucumber recognition model that combined a multipath CNN (MPCNN) with a SVM and color component selection^69^. The detection results showed that the truly classified rate (the rate of pixels correctly classified as true cucumber pixels) for cucumber images was above 90% and the falsely classified rate (the rate of pixels misclassified as true cucumber pixels) was <22%. Quiroz et al.^70^ proposed a model built on a CNN to recognize blueberry plants, and the detection results of the proposed model were as follows: 86% accuracy, 86% precision, 88% recall, and 0.86 F1 score. In another study, Mask R-CNN was applied to improve the detection accuracy of fruits by realizing instance segmentation and the picking point for a strawberry harvesting robot^54^. The detection results from 100 test strawberry images were obtained and are as follows: 95.41% recall, 95.78% precision, and 89.85% mean intersection over union (MIoU). For automatic localization and pose estimation, Giefer et al.^56^ presented an SSD model, named Deep-6DPose (an end-to-end deep-learning framework that recovers a 6D object pose from a single RGB image), that was applied to objects with irregular shapes to establish an automatic fruit grading and packing system.

To acquire accurate and rapid phenotypic trait data, Csillik et al.^71^ and Ampatzidis et al.^72^ combined UAV multispectral imagery with deep-learning methods to detect citrus. The combination of a CNN workflow that utilizes the Google TensorFlow API (https://www.tensorflow.org/api_docs) with a simple linear iterative clustering (SLIC) algorithm was employed by Csillik et al.^71^ and achieved a 96.24% accuracy; the YOLO-V3 model (source code: https://github.com/mystic123/tensorflow-yolo-v3) was used by Ampatzidis et al.^72^ and achieved a 99.8% accuracy. There is another study on methods for classifying cluster crops. As a feature of horticultural products, some products are clustered and should be classified collectively rather than individually. Therefore, a noninvasive DNN classification model for clustered bananas was developed by Le et al.^55^ as a pioneering study based on deep learning for classifying clustered fruits. With the Mask R-CNN model, the proposed deep-learning model reached an overall classification accuracy of 96.5% with only a single side banana image as the input feature.

The techniques for horticultural crop cultivation are somewhat different from those for cultivating other common crops. Regular pruning of crops is needed, and it is also necessary to thin out flowers and fruits to maintain strong trees and to produce higher yields with better quality fruit. Segmenting branches, trunks, flowers and fruits is a key step in automating horticultural cultivation technology. Majeed et al.^20^ adopted a CNN-based semantic segmentation network (SegNet) to detect branch, trunk, and trellis wire to achieve automated tree trimming in an apple orchard. In another study, Sun et al.^73^ used a CNN based on the original Faster R-CNN algorithm to detect and identify flowers and mature (red) and immature (green) fruits of tomatoes. Hu et al.^60^ introduced a method that combined intuitionistic fuzzy set (IFS) theory with the Faster R-CNN model to detect individual ripe tomatoes. The ripe tomato image dataset, which includes adjacent, separated, leaf-shaded, and overlapped images, was used to obtain exact values of the height and width, and these data were then analyzed to evaluate the overall performance of the proposed detection model. Based on the proposed recognition methods, the RMSE results of tomato height and width were 3.306 pixels and 2.996 pixels, respectively.

Although many researchers have conducted a large amount of research on the identification and classification of horticultural products and the proposed methods have high detection performance, modern popular deep neural networks generally require high-performance computing machines for reasoning, which is unrealistic for ordinary farms and orchards.

To reduce the computational cost of networks and meet the computational requirements of real-time devices with low-power-consumption terminal processors, Shi et al.^74^ proposed a generalized attribution method for pruning unnecessary connections in a channel from a well-designed large-scale network to accurately detect mangos in a channel (the source code is available at https://github.com/GlowingHorse/Fast-Mango-Detection). The proposed pruning method can compute the convolutional kernel attributions and fine-tune the network by retaining the important pretrained parameters to extract special mango features. Compared to the YOLO-based network without pruning, the computational cost of the proposed network was reduced by 83.4%, with only an ~2.4% loss in accuracy.

In the examples of the application of machine vision to horticultural crops given above, it can be seen that deep-learning methods have been applied to all aspects of horticultural research, including variety or species classification, key organ classification, and location detection. We also find that researchers’ efforts to apply deep-learning technology to actual production have achieved exciting results in terms of improving performance and detection speed. Table 2 summarizes the technical details of the studies mentioned in this subsection, including the target problems, the compositions of the datasets, the preprocessing methods, the models and frameworks, and the performance comparisons with other algorithms.

**Table 2:** Applications of deep learning in horticultural crop recognition and classification

No	Study	Data	Model used	Performance metric and value	Comparison with other methods	Source code	References
1	Flower classification	OUFD and KLUFD datasets	CNN	CA: 97.78%	Adaboost: 67.81%, ANN:82.45%, DeepANN: 89.74%	Not available	^29^
2	Flower recognition	Flower17 and Flower102 datasets	AlexNet +VGG-16	CA: 96.39% for Flower17 and 95.70% for Flower102	AlexNet: 93.82% for Flower17, 90.90% for Flower102 VGG-16: 92.94% for Flower17, 89.58% for Flower102	Not available	^61^
3	Flower classification	Oxford 102, Oxford 17 and Zou–Nagy datasets	FCN (VGG-16) +CNN	CA: 97.1%, 98.5%, 99.0% on Oxford 102, Oxford 17 and Zou–Nagy, respectively	CNN only: 90.6%, 91.4%, 96.1% on Oxford 102, Oxford 17 and Zou–Nagy datasets, respectively	Not available	^43^
4	Vegetable classification	ImageNet datasets	AlexNet	CA: 92.1%	BP neural network (78%) and SVM classifier (80.5%)	Not available	^30^
5	Cultivar recognition for chrysanthemums	14197 images taken by an RGB camera	ResNet-50	CA: 88.19%	VGG-16:78.21%	Not available	^31^
6	Variety classification for plums	525 images taken by an RGB camera	AlexNet	CA: from 91 to 97% of different plum varieties	NA	Not available	^62^
7	Variety classification for olives	2,800 fruits belonging to seven different olive varieties taken by an RGB camera	Inception-ResNet-V2	CA: 95.91%	AlexNet: 89.90%, Inception V1: 94.86%, Inception V3: 95.33%	Not available	^64^
8	Variety classification for grapes	224 images distributed by six different red grape varieties taken by an RGB camera	AlexNet-based transfer learning scheme	CA: 89.75%	NA	Not available	^63^
9	Variety classification for grapes	35,833 spectra from leaves of 626 plants from 64 varieties taken by a CCD camera	CNN	CA: above 91.63%	SVM: 89.2%	Not available	^65^
10	Fruit detection in apple orchards	800 images from Kinect-v2, 300 images in different scenes were collected	LedNet (LW-Net)	AP: 82.6%, F1: 0.834, Recall: 82.1%, CA: 85.3%	LedNet (ResNet-101): 84.3% (AP), 0.849 (F1), 84.1% (Recall), 86.4% (CA), YOLO-V3: 80.3% (AP), 0.803 (F1), 80.1% (Recall), 82% (CA) YOLO-V3 (Tiny): 78.2% (AP), 0.783 (F1), 77.6% (Recall), 79.6% (CA) Faster-R-CNN (VGG): 81.4% (AP), 0.818 (F1), 81.4% (Recall), 83.5% (CA)	Not available	^67^
11	Apple and branch detection in orchards	800 images from Kinect-v2	DaSNet-B with ResNet-101 backbone	F1: 0.832 on the detection of apples AP: 83.6% inference time: 72 ms	DaSNet-B (LW-Net): 0.821 (F1), 82.7% (AP), 32 ms (time) DaSNet-B (ResNet-50): 0.825 (F1), 83.1% (AP), 47 ms (time) DaSNet-B (DarkNet-53): 0.827 (F1), 83.2% (AP), 50 ms (time)	https://github.com/TensorFlow/models/tree/master/research/slim	^66^
12	Fruit detection for apples	400 RGB-D images and >800 RGB images	DaSNet-V2 with ResNet-101	F1: 0.873, Recall: 86.8%, Precision: 88%	DaSNet-V2 with ResNet-18: 0.857 (F1), 85% (Recall), 87% (Precision) DaSNet-V2 with ResNet-50: 0.868 (F1), 86.1% (Recall), 87.6% (Precision)	Not available	^47^
13	Fruit detection and 3D localization for apples	582 photographs from 1455 apples taken by an RGB camera	Mask R-CNN	F1: 0.816 (2D fruit detection) and 0.881 (3D fruit detection and location)	NA	Not available	^68^
14	Noninvasive classification of bananas	194 banana tier samples with six images from single side images taken by an RGB camera	Mask R-CNN	CA: 96.5%	General machine learning: 79%	Not available	^55^
15	Identification of citrus trees	4,574 UAV multispectral images	CNN (implemented on TensorFlow)	CA: 96.24%, Precision: 94.59%, Recall: 97.94%	NA	Not available	^71^
16	Legacy blueberry recognition	258 images taken by an RGB camera	CNN (implemented on TensorFlow)	CA: 86%, Precision: 86%, Recall: 88% F1: 0.86	NA	Not available	^70^
17	Cucumber recognition	218 effective images taken by an RGB camera	MPCNN combined with RGI and SVM	CA: 97.25%	MPCNN + RGB + SVM: 94.87% CNN + RGB + SVM: 93.36% CNN + RGB + softmax: 92.77%	Not available	^69^
18	Strawberry detection	2000 images taken by an RGB camera	Mask R-CNN (ResNet-50+FPN + RPN)	Precision: 95.78% Recall: 95.41% MIoU: 89.85%	NA	Not available	^54^
19	Tomato detection	800 images taken by an RGB camera	Faster R-CNN combined with IFS	CA: 95.5%, 93.8%, 78.4% and 81.9% of separated, adjacent, overlapped and shaded tomatoes, respectively RMSE: 2.996 pixels for width, 3.306 pixels for height	Faster R-CNN: the RMSE values for the width and height were 7.915 and 8.436 pixels, respectively	https://github.com/rbgirshick/py-faster-rcnn	^60^
20	Mango detection	An open-source mango image dataset	YOLO-V3-tiny network (pruned)	F1: 0.944 GFLOPs: 2.6	YOLO-V3-tiny-512 (without pruning): 0.94 (F1), 8.3 (GFLOPs) Mango-YOLO (a state-of-the-art network trained with the same mango dataset): 0.968 (F1), 15.7 (GFLOPs)	https://github.com/GlowingHorse/Fast-Mango-Detection	^74^
21	Trunk and branch detection for apples	509 RGB images and point cloud datasets by Kinect V2	CNN-based SegNet	Simple-RGB images: 89% (CA), 0.81 (F1) Foreground-RGB images: 94% (CA), 0.92 (F1)	NA	Not available	^20^
22	Key organ detection for tomatoes	5624 RGB images	Faster R-CNN (ResNet-50)	mAP: 90.7%, detection time: 0.073 S/sheet	Original Faster R-CNN (VGG-16): 85.2% (mAP), 0.095 S/sheet (detection time)	Not available	^73^
23	Phenotypic characteristic evaluation for citrus	400 images of 15,000 individual trees from RGB and RNB maps by UAV	YOLO-V3	Precision: 99.9%, Recall: 99.7% F1: 0.998	NA	https://github.com/mystic123/TensorFlow-yolo-v3	^72^
24	Pose estimation for apples	12,280 images	SSD (VGG-16): for the localization of apples Deep-6DPose: for the three-dimensional orientation of an apple	CA: 98.36%	Inception-V3: 91.93% ResNet-50: 95.21% VGG-19: 89.22%	Not available	^56^

### Yield estimate of horticultural crops

An early and accurate estimation of the preharvest yields for horticultural products is generally required by both producers and agricultural companies to guide harvesting and marketing processes for effective and efficient decision making and forward planning. A yield estimation can actually be attributed to the object counting problem in computer vision, which has demonstrated good performance in crowd counting problems^75^ and other plant-related counting problems^76^. In current studies, such yield estimation methods have been adopted for horticultural crops. To estimate a citrus yield, Apolo-Apolo et al.^77^ developed a methodology based on the Faster R-CNN model to detect the existence, count the number and measure the size of citrus fruits and established a network based on LSTM to estimate yields of citrus fruits. The results showed that the average standard error (SE) was only 6.59% when the manual counting result was compared with the proposed model’s detection result. Furthermore, when comparing the actual yields with the estimated yields per tree, the SE was 4.53%, and the standard deviation (SD) was 0.97 kg.

Rebortera et al.^32^ built a deep multifaceted system that incorporated a number of LSTM layers to forecast banana harvest yields. The dataset contained 35,000 observations of banana harvest yields from approximately 2014 to 2018. The enhanced model achieved better performance, which is as follows: the RMSE was 34.805, and the error rates were decreased by 44.95% and 43.5% when compared to models that applied multiple LSTM layers and a single LSTM layer, respectively. In another study, Neupane et al.^78^ developed a deep-learning algorithm (the Faster R-CNN Inception-V2 model) to accurately detect and count bananas in high-resolution RGB images acquired with a UAV^78^. The results showed that 75.8%, 85.1% and 96.4% of bananas were correctly detected on the datasets collected from 60, 50 and 40 meters above ground, respectively, on the same farm, and the recall rate reached 99% when combining the results detected from the 40- and 50-meter datasets. Moreover, as the single-view images from one side of the fruit tree would underestimate the fruit yield since fruit can be hidden by leaves or fruits, a method that applied a video tracking system was proposed and combined MangoYOLO and Kalman filters with the Hungarian algorithm for the detection, tracking, and counting of mango fruits on a tree^79^. Compared to manual counting methods, the proposed video-based tracking model, which added additional imaging perspectives, detected 2050 fruits (62% of the total harvested fruits) with a bias-corrected RMSE of 18.0 fruits per tree, while the two-sided dual-view image model (which also employed MangoYOLO) detected 1322 fruits (40%) with a bias-corrected RMSE of 21.7 fruits per tree. Preharvest yield prediction is also very important for vegetable production. Chen et al.^33^ developed an automatic strawberry flower detection method for predicting yield with a small UAV equipped with an RGB camera. With this system, the mean average precision (mAP) of all detected objects was 83% at 2 m above the ground, and the average accuracy of counting was 84.1%. Rahnemoonfar et al.^80^ proposed a simulated learning method for crop yield estimation by counting fruits based on synthetic tomato images. To capture features on multiple scales, they employed a modified Inception-ResNet model. The detection results showed that the average accuracy was 91% on real images and 93% on synthetic images. In another study, Afonso^81^ adopted the Mask R-CNN model to detect tomatoes in a total of 123 images taken in a greenhouse; the model achieved a better performance than the classical segmentation method. The detection results of the Mask R-CNN model with a ResNeXt-101 architecture showed that the overall precision was 96%, the recall score was 95% and the F1 score was 0.95 for single fruit detection, whereas when the classical segmentation method was used, these parameters were 60%, 80% and 0.69, respectively.

From the above examples of applications in estimating the preharvest yields for horticultural products, we can see that researchers have obtained some good results. With the efforts of scientists, these techniques will be applied to actual production to guide planting plans, harvesting plans and marketing decisions in the future. The technical details of the studies mentioned in this subsection are summarized in Table 3.

**Table 3:** Applications of deep learning in yield estimation of horticultural crops

No.	Study	Data	Model used	Performance metric and value	Comparison with other methods	Source code	References
1	Forecasting banana harvest yields	Thirty-five thousand series of observations from 2014 to 2018.	mdLSTM	RMSE: 34.805	Single LSTM: 61.602 (RMSE)	Not available	^32^
2	Detecting and counting the number of bananas	High-resolution RGB aerial images from 40, 50 and 60 meters above ground	Faster R-CNN Inception-V2	Merging the detection results of the 40- and 50-meter datasets: 99% (Recall), 98.3% (Precision) and 98.7% (CA)	NA	Not available	^78^
3	Predicting the total yield of a commercial citrus orchard.	900 images from a UAV	Faster R-CNN: for fruit detection LSTM: for yield estimation	Between visual counting and the model’s fruit detection: 6.59% (SE) Between the actual and estimated yields per tree: 4.53% (SE), 0.97 kg (SD)	NA	Not available	^77^
4	Video-based mango fruit yield estimation	Video: ~30 frames per tree and a shift of ~20 pixels per frame by the imaging platform.	Video and MangoYOLO-Kalman Filter-Hungarian algorithm method	RMSE: 18.0 fruit/tree	The dual-view image and MangoYOLO-Kalman Filter-Hungarian algorithm method: 21.7 fruit/tree	Not available	^79^
5	Forecast the strawberry yields	The flight images were taken every 2 weeks during the 2017–2018 growing season by a UAV	Faster R-CNN (ResNet-50)	CA: 84.1% mAP: 77.2% Test time: 0.113 s FPS: 8.872	R-CNN (ResNet-50): 61.4% (mAP), 12.024 s (test time), 0.083 (FPS) Fast R-CNN (ResNet-50): 72.3% (mAP), 2.386 s (test time), 0.419 (FPS)	https://arxiv.org/abs/1504.08083	^33^
6	Counting tomato	26400 synthetic images and 100 randomly selected real tomato images from Google Images.	The modified Inception-ResNet	CA: 91% on real images and 93% on synthetic images. RMSE: 2.52 on real images and 1.16 on synthetic images.	Area-based counting: 66.16% Shallow network: 11.6 The original Inception-ResNet-A: 76%	Not available	^80^
7	Detecting and counting the number of tomatoes	A total of 123 images taken by the RealSense camera	Mask R-CNN with ResNeXt-101 architecture	96% (Precision), 95% (Recall), 0.95 (F1)	Classical segmentation: 60% (Precision), 80% (Recall), 0.69 (F1)	https://github.com/wkentaro/labelme	^81^

### Quality detection of horticultural crops

With improvements in living standards, people have increasingly higher expectations for the quality of daily fruits and vegetables. However, fruits and vegetables are susceptible to diseases, insect pests, mechanical damage and improper postharvest treatment during production, planting, storage, transportation, marketing, and other procedures. Both the edible and economic values of horticultural products can be reduced when damage occurs. Therefore, quality detection for horticultural products, especially for fruits and vegetables, is currently a challenging and hot field. In more recent studies, deep-learning methods combined with RGB images or spectrographic techniques have been widely employed as effective and noninvasive horticultural product quality detection approaches to tackle practical problems, including postharvest grading classification, maturity detection, bruise detection, and nutrient content prediction^7^. Compared to traditional machine-learning approaches, deep learning was approaches have been applied to analyze and process image data, spectral data or sound data and have been proven to have better classification performance^82^.

Nasiri et al.^45^ presented a method for discriminating defective dates from healthy dates based on RGB images with a VGG-16 architecture^45^. The introduced CNN model reached a classification accuracy of 96.98%. In another study, Rosanna et al.^83^ explored the application of a deep-learning approach with image processing to classify banana grades and achieved above 90% accuracy. The grading classification of dates and bananas is determined primarily by visible surface defects, and the features can be expressly revealed in RGB image data. However, internal or subdermal damage and the edible quality of fruits and vegetables cannot be recognized visually^1^.

Nondestructive testing technologies, such as near-infrared spectrographs^84^, hyperspectral imagers^85,86^ and thermal imagers^36^, which can detect the internal state of an object without destroying it, have been considered feasible solutions to traditional detection and analysis techniques^82^. Processing large amounts of hyperspectral image data rapidly and accurately is a great challenge. Deep learning, as one of the popular machine-learning methods, has been applied to process complex, massive amounts of data. Wang et al.^82^ focused on the internal and invisible mechanical damage of blueberries utilizing deep-learning technology in combination with hyperspectral transmittance data. The ResNet architecture was chosen for the CNN model. Moreover, five traditional machine-learning algorithms, i.e., linear regression (LR), sequential minimal optimization (SMO), bagging and multilayer perceptron (BMP), and RF algorithms were used for comparison. The fine-tuned ResNet obtained an average accuracy of 88.0% and an F1-score of 0.90. The deep-learning framework has great potential for detecting internal mechanical damage in fruits. In another study, Zhang et al.^85^ applied the deep-learning-based FCN model to the tasks of segmentation and combined it with hyperspectral transmittance images (HSTIs) to accurately detect internal bruising in blueberries. The FCN method achieved better performance than the SVM classifier in both unbruised and bruised fruit prediction. The new full-wavelength method combined with random initialization achieved the best accuracy of 81.2% on the whole test dataset and could be utilized to investigate the resistance of blueberries to mechanical damage and other damage (the source code is available at https://github.com/UGA-BSAIL/BlueberryFCN.git)^85^. Feng et al.^87^ used hyperspectral imaging to detect some minor bruises on winter jujubes. LR, SVM, and CNN models were used for quantitative analyses. The CNN method obtained the highest detection performance, with most of the accuracies exceeding 85%, and the prediction time was also the shortest. The overall performance results revealed the promising and meaningful potential for the detection of minor bruises on winter jujube by utilizing deep-learning methods to analyze the pixelwise NIR spectra and visible and near-infrared (Vis/NIR) spectra collected from hyperspectral imaging systems (the pseudocode of the method was given in the original paper). Liu et al.^86^ developed a novel classification method by applying the combination of a stacked sparse autoencoder (SSAE) with a CNN to detect defects in pickling cucumbers with a hyperspectral imaging-based dataset. The results showed that, compared with the SSAE model, the CNN-SSAE method improved the performance of a six-class classification task and achieved overall accuracies of 88.3% and 91.1% at conveyor speeds of 165 and 85 mm s^-1^, respectively.

SSC and firmness are the most significant properties of edible quality in horticultural products and directly influence consumer satisfaction^27^. The Vis/NIR hyperspectral imaging technique has been used for the nondestructive detection of the internal quality attributes and the chemical composition in fruits due to its high sensitivity and accuracy. The principle behind the spectral detection method is to measure the spectrum of reflected or transmitted missions from fruit and to construct a relationship between the measured spectrum and the chemical composition of the fruit. The received spectrum can then be used to indicate the SSC and/or firmness by referring to some chemometric methods^27^.

Bai et al.^84^ focused on the accurate prediction of SSC in apples collected from a number of geographical origins. A multiorigin SSC prediction method for apples was developed by the combination of NIR analysis, spectral fingerprint feature extraction, optimal wavelength selection, model search strategies, origin recognition, and multivariate regression analysis with deep learning. The correlation coefficients of prediction (R_P_) and RMSEs of prediction (RMSEP) values of 99.0% and 27.4%, respectively, were obtained by the proposed model. In another study, Yu et al.^27^ developed a deep-learning method consisting of SAE and FNN coupled with a hyperspectral imaging technique for the prediction of SSC and the firmness of postharvest pears. The proposed model obtained reasonable prediction performance with coefficients of determination of the prediction set (*R*^2^_P_) = 92.1%, the RMSEP = 0.22% and the ratio of the prediction to the deviation of the prediction set (RPD_P_) = 3.68 for SSC, and *R*^2^_P_ = 89.0%, RMSEP = 1.81 N and RPD_P_ = 3.05 for firmness.

The laser backscattering method is another optical technique that can be used for the nondestructive detection of fruit samples. Wu et al.^34^ constructed an AlexNet model with an 11-layer structure, identified the defect, normal, stem and calyx regions of apples with laser-induced light backscattering imaging, and achieved a higher recognition rate of 92.5% and an accuracy better than those obtained by conventional machine-learning algorithms.

In addition to spectroscopy technology, acoustic sensing is also a reliable method for the nondestructive detection of horticultural products. Lashgari et al.^88^ applied acoustic and deep-learning techniques to detect mealy and nonmealy apples. VGGNet and AlexNet, which are both famous pretrained CNN models, were used to classify the apples. The accuracies of VGGNet and AlexNet for classifying mealy and nonmealy apples were 86.94% and 91.11%, respectively. Although VGGNet is deeper and performed better on ImageNet (see Table 1), in combination with an acoustic sensing system, AlexNet had a superior ability in terms of classification accuracy, training and classification speed compared to VGGNet in this particular work.

In commercial orchards, it is important to monitor the maturity of fruit during the whole development period to determine the optimal time to harvest. Automated machine vision techniques are widely used to detect and identify the growth and maturity stages. Wendel et al.^8^ proposed a novel approach that utilized a LIDAR sensor, a hyperspectral camera and a navigation system fixed to a ground vehicle for predicting the dry matter (DM) content of individual fruit from a commercial mango orchard. A cross-validation *R*^2^_CV_ = 64% and RMSE_CV_ = 1.08% w/w were achieved by the CNN for fruit on trees, while a *R*^2^_CV_ = 58% and RMSE_CV_ = 1.17% w/w were achieved by PLS. In another study, a CNN was used to evaluate citrus maturity by utilizing a fluorescent spectrum signal^89^. They adopted fluorescence spectroscopy to estimate the Brix/acid ratio. As a result, the absolute error of the Brix/acid ratio was 2.48, which was significantly better than the values achieved by other previous methods.

These investigations showed that some physical and chemical properties of fruits and vegetables (including nutrient content, hardness, degree of damage, degree of disease, and degree of maturity) can be revealed through RGB images, sample spectral information and acoustic spectral information. Better prediction and classification effects can be achieved through deep-learning model training. In Table 4, we summarize the technical details of the studies mentioned in this subsection.

**Table 4:** Applications of deep learning in the quality detection of horticultural products

No.	Study	Data	Model used	Performance metric and value	Comparison with other methods	Source code	References
1	Discriminating healthy date fruit from defective ones	Over 1300 images of healthy and defective dates by LG-V20 cell phone camera	The modified CNN (VGG-16)	CA: 96.98%	Outperforms the traditional classification methods	Not available	^45^
2	Postharvest grading classification of Cavendish bananas by surface defects and finger size	1116 images including the front and back surface of the banana	CNN	CA: 93%	NA	Not available	^83^
3	Detection of internal mechanical damage of blueberries	Hyperspectral transmittance image of 737 blueberries from December 2014 to January 2015	The fine-tuned ResNet and the improved version named ResNeXt	ResNet: 88.44% (CA), 93.25% (Recall), 86.23% (Precision), 0.8985 (F1) ResNeXt: 87.84% (CA), 89.44% (Recall), 88.67% (Precision), 0.8905 (F1)	SMO: 80.82% (CA), 92.16% (Recall), 76.07% (Precision), 0.8286 (F1) Linear Regression: 76.06% (CA), 83.28% (Recall), 74.03% (Precision),0.7796 (F1) RF: 73.14% (CA), 80.31% (Recall), 71.48% (Precision),0.7529 (F1) Bagging: 71.13% (CA), 77.51% (Recall), 69.99% (Precision),0.7339 (F1) Multilayer Perceptron: 78.27% (CA), 85.52% (Recall), 75.23% (Precision), 0.7971 (F1)	Not available	^82^
4	Early detection of internal bruises of blueberries	A total of 1200 images collected from hyperspectral transmittance imaging	The fine-tuned FCN model (87-newModel: use all the wavelengths)	CA: 81.2% for unbruised, 77.8% for bruised and 84.5% for calyx	SVM: 67.9% for unbruised, 49.4% for bruised and 22.5% for calyx	https://github.com/UGA-BSAIL/BlueberryFCN.git	^85^
5	Detection of subtle bruises of winter jujubes	150 samples of single geographical origin (a total of 4) were collected, and the data were acquired by NIR and Vis-NIR hyperspectral imaging system	CNN	On all geographical origin and using NIR spectra: 99.62-100% (CA), 0.59 s (computation time)	SVM: 98.54-100% (CA), 7.25 s (computation time) LR: 97.78% (CA), 0.37 s (computation time)	Pseudocode provided in the reference.	^87^
6	Detecting internal and external defects of cucumbers	A total of 230 samples with five classes were tested, Hyperspectral imaging system	CNN-SSAE	CA: 91.1% and 88.3% at conveyor speeds of 85 mm/s and 165 mm/s, respectively	SSAE: 88.0% and 86.7% at conveyor speeds of 85 mm/s and 165 mm/s, respectively	Not available	^86^
7	Detection of apple defects	Laser backscattering spectroscopic images of apples (A total of 500 samples) are obtained using semiconductor laser	AlexNet	CA: 92.5%	The CA is better than conventional machine-learning algorithms (BP, SVM, and PSO algorithm)	Not available	^34^
8	Prediction of SSC of apples and origin recognition	FT–NIR spectra data and spectral fingerprint features (A total of 208’ Fuji’ apples)	AEs coupled with spectral fingerprint features	R_P_: 99.0% RMSEP: 27.4%	Compared with the individual-origin model, the proposed multiple-origin model achieved more accurate results for the prediction of SSC of apples	Not available	^84^
9	Predicting firmness and SSC of Korla fragrant pears	Vis/NIR hyperspectral reflectance imaging (a total of 180 pear fruit)	SAE-FNN	*R*^2^_P_: 0.890 and RMSEP: 1.81 N for firmness *R*^2^_P_: 0.921 and RMSEP: 0.22% for SSC	PLSR: *R*^2^_P_: 0.842 and RMSEP: 2.17 N for firmness *R*^2^_P_:0.832 and RMSEP: 0.33% for SSC LS-SVM: *R*^2^_P_: 0.795 and RMSEP: 2.47 N for firmness *R*^2^_P_: 0.784 and RMSEP: 0.37% for SSC	Not available	^27^
10	Detection of mealiness of apple	Acoustic sensing system (A total of 180 samples)	AlexNet and VGGNet	CA: 91.11% of AlexNet	CA: 86.94% of VGGNet	Not available	^88^
11	Maturity estimation of mangos	Hyperspectral imaging (A total of 78 fruit)	CNN	*R*^2^_CV_: 64% and RMSE_CV_: 1.08% w/w	PLS: *R*^2^_CV_: 58% and RMSE_CV_: 1.17% w/w	Not available	^8^
12	Estimation of citrus maturity (the Brix/acid ratio)	Fluorescence spectroscopy (47 citrus fruits)	CNN regression	AE: 2.48	Principal Component Regression: 4.04 (AE)	Not available	^89^
13	Detection and classification of pear bruises	Thermal images (3246 samples) collected from 300 pears over 10 days	LeNet (with the Adam optimizer)	CA: 99.25%	LeNet (with the gradient descent optimizer): 76.3% LeNet (with the Adagrad optimizer): 96%	Not available	^36^

### Detection of biotic/abiotic stress in horticultural crops

Singh et al.^6^ reviewed the application of deep-learning methods to plant stress phenotypes in 2018. According to their summary, deep learning can be applied to the identification, classification, quantification, and prediction (also called the ICQP paradigm) of plant stress phenotyping^6^. In this section, we comprehensively outline the publications that employ deep learning for the stress phenotyping of horticultural plants (Table 5). The traditional identification and classification of plant stress have always relied on the recognition of visual symptoms by human experts as a means of categorization, which is inevitably subjective and error prone^90^. Computer vision coupled with machine-learning technology has the capability of automatic identification and classification and enables accurate, scalable and high-throughput phenotyping. Among the machine-learning methods, deep learning has been considered one of the most effective approaches for improving the overall performance of object detection and recognition processes^6,91^.

**Table 5:** Applications of deep learning for the detection of stress in horticultural crops

No.	Study	Stress name	Sensor	Model used	Performance metric and value	Comparison with other methods	Source code	References
1	Disease and pest recognition of tomatoes	Nine classes (Leaf mold, Gray mold, Canker, Plague, Miner, Low temperature, Powdery mildew, Whitefly, Nutritional excess)	RGB images	Faster R-CNN (VGG-16), Faster R-CNN (ResNet-50)	Faster R-CNN (VGG-16): 83% (mAP) Faster R-CNN (ResNet-50): 75.37% (mAP) Faster R-CNN (ResNeXt-50): 71.1% (mAP)	SSD (ResNet-50): 82.53% (mAP) R-FCN (ResNet-50): 85.98% (mAP)	Not available	^91^
2	Disease detection of tomatoes	Three diseases (early blight, late blight, and leaf mold)	RGB images	Attention embedded residual CNN	CA: 98%	SVM: 92% LeNet based CNN: 95% VGGNet based CNN: 95.24% ResNet: 97.28%	Not available	^92^
3	Disease detection of tomatoes	Nine diseases (Tomato yellow leaf curl virus, Tomato mosaic virus, Target spot, Spider mites, Septoria spot, Leaf mold, Lateblight, Earlyblight, Bacterial spot)	RGB images	AlexNet and GoogLeNet	CA: 98.66% in AlexNet CA: 99.18% in GoogLeNet	SVM: 94.54% RF: 95.47%	Not available	^46^
4	Disease classification of *Solanum melongena*	Five diseases (*Epilachna* beetle infestation, little leaf, *Cercospora* leaf spot, two-spotted spider mite and Tobacco Mosaic Virus (TMV))	RGB images	The modified VGG-16	CA: 93.3%	VGG-16: 90% AlexNet: 46.66% Modified AlexNet: 60%	Not available	^94^
5	Diseases recognition of cucumbers	Four diseases (anthracnose, downy mildew, powdery mildew, and target leaf spots)	RGB images	AlexNet	CA: 93.4%	RF: 81.9% SVM: 84.8%	Not available	^93^
6	Disease identification of cucumbers	Six diseases (downy mildew, anthracnose, gray mold, angular leaf spot, black spot, and powdery mildew)	RGB images	GPDCNN (based on AlexNet)	CA: 94.65% Testing time: 3.58 s	Classical CNN: 91.73% (CA), 3.64 s (testing time) AlexNet: 92.48% (CA), 3.72 s (testing time)	Not available	^95^
7	Segmentation and quantification of cucumber disease	Cucumber powdery mildew	RGB images	CNN based on the U-Net	CA (Pixel): 96.08%, Precision: 73.3% Recall: 97.34	*K*-means: 92.33% (CA), 71.35% (Precision), 60.55% (Recall) RF: 93.95% (CA), 70.99% (Precision), 69.33% (Recall)	https://github.com/ChrisLinSJTU/segmentation-of-powdery-mildew	^35^
8	Disease identification of onions	Onion downy mildew	RGB images	CNN (based on VGG-16)	The mAP at IoU criteria 0.5 range from 74.1 to 87.2.	NA	Not available	^96^
9	Disease identification of potatoes	Four diseases (black dot patches, black scurf patches, common scab patches, silver scurf patches)	RGB images	CNN (based on VGGNet)	CA: 96%	NA	Not available	^97^
10	Disease identification of mangos	Anthracnose disease	RGB images	MCNN	CA: 97.13%	PSO: 88.39% SVM: 92.75% RBFNN: 94.20%	Not available	^42^
11	Disease identification of bananas	Three disease (healthy, black sigatoka and black speckle)	RGB images	LeNet	CA: 99.72% Precision: 99.70% Recall: 99.72% F1: 0.9971	NA	Not available	^41^
12	Disease identification of apples	Six diseases (healthy apple, general apple scab, serious apple scab, apple gray spot, general cedar apple rust and serious cedar apple rust)	RGB images	Based on DenseNet-121	CA: 93.31%, F1: 0.9339	The traditional multiclassification method based on cross-entropy loss function: 92.29% (CA), 0.9228 (F1)	Not available	^99^
13	Disease identification of olives	Six diseases (Anthracnose, Canker, Lepra Fruit Rot, Peacock Spot, Parlatoria Oleae, Aspidiotus Nerii)	RGB images	The modified AlexNet	CA: 99.11% Precision: 99.49% Recall: 99.11% F1: 0.9929	AlexNet: 97.88% (CA), 97.42% (Precision), 97.37% (Recall), 0.9736 (F1)	Not available	^98^
14	Classification and Discrimination of peach diseases	The fungal diseased peaches in the slight, moderate or severe level.	Hyperspectral images	DBN	based on the combined features: CA: 93.3%, 100%, and 100% for slightly, moderately and severely decayed samples, respectively	PLSDA: based on the combined features: CA: 88.3%, 100%, and 100% for slightly, moderately and severely decayed samples, respectively	Not available	^100^
15	Disease identification of tulips	Tulip Breaking Virus (TBV)	RGB-NIR images	Faster R-CNN	Precision: 52% Recall: 86% F1: 0.65	Crop experts: 81% (Precision), 68% (Recall), 0.74 (F1)	https://github.com/rbgirshick/py-faster-rcnn	^59^

To identify various biotic and abiotic stresses in tomatoes, efforts have been made with different kinds of deep-learning approaches. Fuentes et al.^91^ combined three deep-learning meta-architectures, namely the Faster R-CNN, region-based FCN (R-FCN), and SSD, with two deep feature extractors (ResNet and VGGNet) to detect pests and diseases in tomatoes. To detect diseases in tomatoes, Karthik et al.^92^ proposed a model to apply an attention gating mechanism in a residual CNN with the PlantVillage dataset, which contains three kinds of diseases in tomatoes, namely, leaf mold, early blight, and late blight, for disease detection. An overall accuracy of 98% was achieved with the proposed model on the validation datasets by adopting a fivefold cross-validation method, in which the original sample was randomly divided into five subsets of equal size and one subset was used as the validation data and the other four for training the model. The cross-validation process was then repeated five times, and the results from the five iterations were averaged (or otherwise combined) to produce a single estimation. In another study, images of tomatoes were acquired from the open PlantVillage database; the images included various bacterial (bacterial spot), viral (tomato mosaic virus and yellow leaf curl virus), and fungal (leaf mold, target spot, early blight, and late blight) diseases and pests (such as spider mites). The proposed framework reached an accuracy of 99.18% with GoogLeNet, while AlexNet had an accuracy of 98.66%^46^.

For the identification of various diseases in cucumbers (diseases such as anthracnose, powdery mildew, downy mildew, gray mold, target leaf spots and black spot), a deep-learning approach was also used. Lin et al.^35^ presented a semantic segmentation method based on a CNN to identify powdery mildew on cucumber leaves and achieved the following results: the average pixel accuracy was 96.08%, the Dice accuracy was 83.45% and the intersection over union was 72.11% (the source code is available at https://github.com/ChrisLinSJTU/segmentation-of-powdery-mildew)^35^. In another study on a recognition model for cucumber diseases, the DCNN model achieved an accuracy of 93.4%^93^.

In another application, AlexNet and VGG-16 were proposed to classify five eggplant diseases (little leaf, epilachna beetle infestation, cercospora leaf spot, tobacco mosaic virus (TMV) and two-spotted spider mite) and healthy plants with images acquired from smartphones. They used the modified VGG-16 model to achieve an accuracy of 93.33%^94^. Another novel deep-learning architecture with a global pooling dilated CNN (GPDCNN) was presented for cucumber leaf disease (gray mold, powdery mildew, anthracnose, downy mildew, black spot, and angular leaf spot) recognition by the combination of a dilated convolutional neural network with global pooling^95^. The results showed that GPDCNN had a higher recognition accuracy and shorter training time than the DCNNs and AlexNet^95^.

In a recent study, an image-based field monitoring system combined with a weakly supervised training method was developed for automatic onion disease detection and growth monitoring in real-time. The results showed that the mAP (mean Average Precision) score at an IoU (Intersection of Union) criteria of 50%, which indicates a 50% overlap, was the highest among all the existing models and was between 74.1 and 87.2^96^. In another study, a CNN was trained to classify diseased potato tubers into five classes, including four disease classes and a healthy class, with an accuracy of 96%^97^. Alruwaili et al.^98^ proposed an enhanced CNN model named AlexNet for detecting and classifying olive diseases. The proposed method achieved overall accuracy, recall, precision, and F1 scores of 99.11%, 99.11%, 99.49%, and 0.9929%, respectively. In another application, three methods, including regression, multilabel classification and a focus loss function based on the DenseNet-121 DCNN, were proposed to detect diseases on apple leaves^99^. The proposed three methods obtained accuracies of 93.5%, 93.3%, and 93.7% on the test dataset, which are better than those obtained by the traditional multiclassification approach.

With the goal of automating disease identification and classification, a multilayer CNN (MCNN) was proposed for classifying mango leaves infected by anthracnose fungal disease with an accuracy of 97.13%^42^. The LeNet architecture was applied to classify and identify banana leaf diseases^41^. The Faster R-CNN architecture was proposed to automatically detect the Tulip Breaking Virus (TBV) and reached an efficiency of 0.13 s per image^59^. A deep belief network (DBN) model based on 494 features was developed to classify peach samples with slightly decayed, moderately decayed and severely decayed diseases. The results showed that the highest classification accuracies for the three kinds of peach diseases mentioned above were 82.5%, 92.5%, and 100%, respectively^100^.

The early and accurate detection of plant diseases is considered an effective method to maintain and improve crop quality and minimize production losses. As a result, deep-learning approaches have received wide recognition worldwide because of their accurate and efficient detection of plant diseases in the field. In Table 5, we summarize the technical details of the studies mentioned in this subsection.

### Growth monitoring in horticultural crops

Crop traits are important to plant breeders and producers for plant production management, as well as for making intelligent decisions about excellent genotype selection when yield traits or quality traits are used. The automatic intelligent collection of horticultural crop growth information in advance provides a good basis for planters to monitor growth and plan the harvesting timeline during the maturation of fruits and vegetables^37,48^. Lu and his team have proposed methods to localize mushrooms and track their growth^37,57^. Lu et al.^57^ adopted the YOLO algorithm to localize mushrooms in an image and proposed a positioning correction method to modify the localization result. After the mushrooms had been precisely localized, Lu et al.^37^ developed an image measurement system to record the diameter of the mushroom caps during the maturation period. The proposed algorithm (the YOLO-V3 + SP algorithm) was used to calculate the mushroom circles based on the images captured by a camera continuously and then to record the growth information of the mushroom caps; the method outperformed the current circle Hough transform method (OpenCV’s implementation)^37^.

Automatic detection and identification of fruits and flowers at various growth stages is important for the automatic and intelligent management of orchards. Tian et al.^101^ proposed an instance segmentation model by improving the mask scoring R-CNN with a U-Net backbone (MASU R-CNN) to detect and segment apple flowers at three different stages: bud, semiopen and fully open. Furthermore, Tian et al.^102^ proposed an improved YOLO-V3 model to detect apples at various growth stages, i.e., young, expanding, and ripe apples, in orchards with complex backgrounds. The detection performance of the proposed YOLO-V3-dense model was better than that of the original YOLO-V3 and the Faster R-CNN with VGG-16 net models. Wang et al.^48^ also developed an automated growth monitoring system in an apple orchard to monitor apple growth during the period of fruit thinning and fruit ripening. They used the fused convolutional features (FCF) model to segment apple images. The mean average absolute error of an apples’ horizontal diameter obtained by the method was 0.90 mm. In another study, Tu et al.^44^ developed a machine vision model for detecting passion fruits and identifying their maturity by utilizing natural outdoor RGB-D images combined with the DSIFT (dense scale invariant features transform) algorithm and the LLC (locality-constrained linear coding) method. Finally, the features collected by RGB-DSIFT-LLC were fed into a SVM classifier for the identification of fruit maturity at five different levels: young (Y), near-young (NY), near-mature (NM), mature (M), and after-mature (AM). The proposed method achieved an accuracy of 92.7% for detection and 91.5% for maturity classification. Ni et al.^38^ adopted another deep-learning method based on the Mask R-CNN model and an iterative annotation strategy to detect and segment blueberry fruit to monitor the maturity of the blueberry fruit. The proposed model obtained reasonable prediction performance, with a coefficient of determination (*R*^2^) for the detected berry number with respect to the ground truth of 88.6% and a RMSE of 1.484.

It is important to recognize the different sizes of the panicle-associated image area and the number of panicles as indices of flowering. Wang et al.^103^ developed a machine vision assessment system to detect flower panicles on mango plants at different stages of growth, from green to light yellow, light pink, and then brown-red, which correspond to panicle development ranging from the early to late stages. In another study, Koirala et al.^104^ proposed four architectures based on a deep-learning method for mango panicle stage classification at three different stages: panicles with flowers not fully opened (whitish in color), panicles with opened flowers and panicles displaying flower drop and fruit set. While the YOLO-V3-rotated model had a higher accuracy in terms of the total number of panicles, the *R*^2^CNN-upright model was superior for the classification of panicle stages (the source code for the *R*^2^CNN method is available at: https://github.com/DetectionTeamUCAS/R2CNN_Faster-RCNN_Tensorflow)^104^. To extract growth and quantity information from the large-scale aerial images collected from a lettuce field, Bauer et al.^105^ proposed the AirSurf-Lettuce platform, which applied a CNN-based-learning model (the source code is available at https://github.com/Crop-Phenomics-Group/AirSurf-Lettuce). The AirSurf-Lettuce can automatically measure in-field iceberg lettuces with a focus on yield-related traits, such as field size distribution, lettuce size categories, number of plants, and GPS-tagged harvest regions, and has great potential to support smart and precise crop surveillance.

From the investigations given above, it is known that deep-learning methods have been applied to growth monitoring in horticultural crops and have achieved better prediction and classification effects, the technical details of which are summarized in Table 6.

**Table 6:** Applications of deep learning in growth monitoring of horticultural crops

No.	Study	Data	Model used	Performance metric and value	Comparison with other methods	Source code	References
1	Measurement the circle diameter of common mushroom caps	The image measurement system automatically measures the growth of the common mushrooms and calculated the data every hour	YOLO-V3 + SP algorithm (The proposed algorithm employed YOLO-V3 for image positioning and SP algorithm to estimate common mushroom circles)	The average value of Op (Larger Op means higher accuracy of the circle in the round mushroom caps) is 82.7%, Oq (represents the deviation of the algorithm in circle detection) is 4.4%	CHT method (OpenCV’s implementation): Op (average 43.8%), Oq (average 19.1%)	Not available	^37^
2	To measure the mushroom size and to count the number of mushrooms.	A smart mushroom measurement system	YOLO-V3	The average error of estimated and known harvest time was 3.7 h.	NA	http://pjreddie.com/yolo/	^57^
3	Automatic detection of apple flowers and fruits at different growth stages	The apple flower images were collected in an orchard (a total of 600 images)	MASU R-CNN	Precision: 96.43%, Recall: 95.37%, F1: 0.9590, mAP: 59.4%	CNN + SVM: 92.7% (Precision), 92.0% (Recall), 0.934 (F1)	Not available	^101^
4	Detecting apples during different growth stages in orchards	Image acquisition was conducted with a camera during different growth stages (a total of 480 images)	YOLO-V3-dense	F1: 0.817, The average detection time is 0.304 s per frame.	YOLO-V2: 0.738 (F1), 0.273 s (detection time) YOLO-V3: 0.793 (F1), 0.296 s (detection time) Faster R-CNN (VGG-16): 0.801 (F1), 2.42 s (detection time)	Not available	^102^
5	Detection to remotely monitor apple growth	Images captured by the remote apple growth monitoring system (A total of 21 apples were monitored)	FCF network (based on ResNet-50)	F1: 0.531 The average GPU running time was 0.075 s per image	The mean average AE of the apples’ horizontal diameters is 0.90 mm, which decreased by 67.9% comparing to the circle fitting-based method (2.8 mm)	Not available	^48^
6	Maturity classification of passion fruits from five categories: young (Y), near-young (NY), near-mature (NM), mature (M) and after-mature (AM).	RGB-D images were obtained from a passion fruit farm by a Kinect V2.0 device (overall 4000 images)	Faster R-CNN (VGG-16) for detection SVM for classification RGB-DSIFT-LLC for feature extraction	Recall: 84.49% CA: 91.52%	Red-DSIFT: (78.4% CA) Green-DSIFT: (82.52% CA) Blue-DSIFT: (76.9% CA) RG-DSIFT: (87.6% CA)	https://github.com/shaoqingren/faster_rcnn	^44^
7	Panicle stage classification of mangos	The image set of 994 trees from orchard A and 24 trees from orchard B by RGB camera	YOLO-V3-rotated (for panicle count)and *R*^2^CNN-upright (for panicle stage classification)	YOLO-V3- rotated: 15.4 (RMSEs), 65.0% (mAP), 0.749 (F1) *R*^2^CNN-upright: 32.3 (RMSEs), 70.9% (mAP), 0.820 (F1)	MangoYOLO (upright): 25.6 (RMSEs), 72.2% (mAP), 0.765 (F1) MangoYOLO-rotated: 16.0 (RMSEs), 69.1% (mAP), 0.761 (F1) *R*^2^CNN (rotated): 25.8 (RMSEs), 62.5% (mAP), 0.740 (F1)	https://github.com/DetectionTeamUCAS/R2CNN_Faster-RCNN_TensorFlow	^104^
8	Assessment of mango orchard flowering	Imagery of mango tree canopies was acquired from two commercial orchards by two imaging platforms	Faster R-CNN	*R*^2^: 86% RMSE: 30.1 between two replicate human counts of panicles per trees	The highest correlation was achieved against manual counts.	Not available	^103^
9	Monitor lettuce growth from large-scale aerial images collected from the field	The ultralarge aerial NDVI imagery was acquired	CNN-based	CA: >98%	The correlation between the human and automatic counting is ~2% (*R*^2^ = 0.978) for the small regions, and 0.8% (*R*^2^ = 0.988) for the large regions	https://github.com/Crop-Phenomics-Group/AirSurf-Lettuce	^105^
10	Count berries, measure maturity, and evaluate compactness of blueberries	The Blueberry images were obtained under three different lighting conditions and backgrounds (a total of 669 images)	Mask R-CNN	mAP: 78.3% for the validation and 71.6% for test dataset under 0.5 IoU threshold and corresponding mask accuracy: 90.6% for the validation and 90.4% for test dataset	The *R*^2^ value between the detected berry number and the ground truth is 0.886 with a root-mean square error (RMSE) of 1.484	Not available	^38^

### Other applications

Genomic prediction (GP) is the process in which untested good genetic attributes are predicted by employing genome-wide marker information^40^. Recently, deep-learning technologies have been applied as powerful machine-learning tools to quantitatively predict phenotypes without intrudation to analyze the increasing amount of available genetic and genomic data. Although numerous examples of GP have been widely utilized to improve the breeding efficiency of plants and animals^40,106^, applications to horticultural crops are still in the preliminary stage. Zingaretti et al.^40^ evaluated the genomic prediction performance of a deep-learning method for two common and important horticultural fruits: the autotetraploid blueberry and allooctoploid strawberry. The two datasets included a total of 1802 autopolyploid blueberry (2*n* = 4*x* = 48) and 1358 allopolyploid strawberry (2*n* = 8*x* = 112) individuals genotyped to create 73,045 and 9908 single-nucleotide polymorphism (SNP) markers, respectively, and phenotyped by five distinctive agronomic traits, including fruit size, firmness, picking scars, weight, and yield. A potential superiority of deep learning for GP over some standard linear approaches is that deep learning can potentially consider all possible genetic interactions, including epistasis and dominance, which are considered to be particularly relevant in most polyploids.

The sale of horticultural products is an essential part of the product supply chain. Owing to the asymmetry between a farmer’s production and the real marketing information and the asymmetry between the social signal of product supply and demand, the prices of horticultural products fluctuate greatly. Therefore, it is very important to forecast the prices of horticultural products when creating a planting plan^39^. Weng et al.^39^ adopted the autoregressive integrated moving average (ARIMA), BP and RNN methods to forecast the daily, weekly, and monthly average prices of different horticultural products (cucumbers, tomatoes, and eggplants). With web crawler technology, a large amount of data on horticultural product prices were gathered from the website. The results indicated that neural network methods including the BP and RNN methods had a higher accuracy in price forecasting than that of the ARIMA model. They considered that deep-learning methods would serve as the mainstream method for price forecasting of horticultural products in the near future.

## The advantages and disadvantages of deep-learning technology

The most notable advantages of deep-learning technologies lie in their automatic feature extraction, classification and prediction processes^7^. The handcrafted extraction of features and the design of feature descriptors are generally very difficult and time consuming and are no longer required for deep-learning technologies through automated feature extraction. Generally, the prediction accuracy of deep-learning models improves as the number of model layers increases, which is accompanied by increased computational complexity. In addition, it is not an easy task to build a good feature extractor. In fact, a great deal of the aforementioned studies for the prediction and classification of horticultural plants, including flowers or fruits, used existing models or made only a minor adjustment to them, and the main contributors were also mostly scholars from computer science and image processing sectors. Therefore, higher attention was given to the techniques for general feature extraction and classification of images rather than the specific features of a horticultural product. For instance, in the applications of crop yield estimation, the manual extraction of features that may significantly affect crop growth was nearly impossible. Thus, it is important to design deep-learning models that specifically focus on such feature extraction processes to more efficiently and cost-effectively apply deep-learning models to horticultural sciences. Fortunately, deep learning has another useful characteristic: its transfer learning ability^28^. With this technique, researchers can make use of the existing models already trained by a large amount of source data to solve similar problems. By doing so, they generally need to adjust some layers and use only the target data (the data they are going to learn) to fine-tune the already trained model. Through fine-tuning, the efficiency and performance in modeling subsequent tasks is improved. For example, in the studies mentioned above, some examples exploited predesigned networks (e.g., VGGNet or AlexNet) based on a large dataset (such as ImageNet) and applied them to their specific learning task that required a much smaller dataset through fine-tuning to achieve better-than-before results.

As deep-learning contains more complex model structures and requires higher computational efforts, its development and applications are somewhat limited for noncomputer experts^7^. Fortunately, contributors, such as computer scientists and deep-learning enthusiasts, around the world have developed many software and hardware tools to help nonexpert researchers to easily and quickly develop deep-learning technology. For software support, some popular models and their variations have been designed to reduce programming difficulties so that nonexpert researchers can build required networks quickly. The aforementioned models, such as AlexNet, CNN-SSAE, DaSNet, LedNet, and VGGNet, are just a few. Apart from these ready-to-use models, there are also a number of websites from which researchers in horticulture sectors can learn to improve their deep-learning skills and build their own models. For example, https://www.fast.ai/ provides online courses that are free and have no ads, https://www.deeplearning.ai/ provides specialized deep-learning techniques; AI for everyone, TensorFlow specialization, and https://mlcourse.ai/ provide open machine-learning courses. For hardware support, a GPU combined with the compute unified device architecture (CUDA) toolkit developed by NVIDIA is a good candidate to accelerate deep-learning computation. There is also some specialized hardware designed for accelerating deep-learning processes, among which the Tensor Processing Unit (TPU) developed by Google, the AI Processors, Vision Processing Units (VPUs) and the Neural Compute Stick 2 (NCS 2) by Intel, the Efficient Inference Engine (EIE) by Stanford, and the Energy-Efficient Reconfigurable Accelerator for DCNNs (Eyeriss) by MIT are some useful tools. The proposed network frameworks and hardware acceleration tools can be used together, can greatly reduce the computational time and have the potential to perform prediction and/or classification well to meet the needs of real-time applications in data processing.

Nevertheless, it cannot be denied that deep learning has its own shortcomings. First, the optimization tasks are sometimes quite complicated and very time consuming due to the large datasets and large numbers of weights to be tuned; additionally, there are some hardware restrictions and numerous hyperparameters, which are highly complex, that need to be tuned in the models mentioned in Section “Brief overview of deep learning”. Furthermore, to replicate a given study and compare it with others, the source codes/algorithms and the model parameters must be reported, and the evaluation metrics for the performance measures should be standardized. From the reported studies, regarding the methods used in the different research works, various performance metrics have been employed by the authors. For example, we noted that performance metrics, such as accuracy, precision, recall, F1-score, mAP, RMSE and IoU, were employed in the literature to report model performance. However, to compare a reported model and to improve model performance in the existing studies, the use of performance metrics should be unified and standardized in future research. Additionally, different models, learning algorithms, hyperparameters or validation processes have been applied and vary from species to species or object to object. Such specificity limits the widespread application of the proposed technologies outside of the given research domain. Further work should be done to standardize the proposed technologies and overcome the bottleneck to build robust and easy-to-use models. Noncomputer experts, such as horticulture scientists, will be able to find and use such user-friendly models for practical applications in the future.

Another limitation lies in the fact that the collection of a large and reliable dataset along with clear data annotation is inevitably tedious work, which make completing tasks more complicated for researchers. The success of deep learning cannot be realized without the availability of annotated data^107^. Data collection and annotation is crucial and time consuming; therefore, data collection efforts should be made by researchers worldwide. Moreover, since the collection ability of data by individuals, research teams, or even institutions is limited, the collected data should be uploaded to open-access databases to lower the entry barrier and to accelerate the availability of of data for researchers. On the other hand, a number of ongoing examples of code sharing are now available on GitHub and other Git-like platforms developed by some enthusiasts for nonexperts to share models and check data^1^. Therefore, a combined effort by both horticulture and computer scientists is necessary to make significant contributions to meet challenges in intelligent horticulture fields.

## Summary, discussions, and future perspectives

### Summary and discussions

Through a careful examination of existing studies, we found that the major research focus was on the development of deep-learning models and their potential applications in various horticultural studies. The application of deep-learning techniques in horticultural sectors is still in its nascent stage but is also in a period of rapid development. From 2016 until now, there have been 71 relevant publications focusing on the applications of deep-learning methods to species identification and variety classification (33.80%), quality detection (18.31%), yield prediction (9.86%), pest and disease management (21.13%), and growth monitoring (14.08%) in the field of horticultural crops in addition to some review papers (2.82%). Among the surveyed papers, the number of publications in 2019 was 130% more than that in 2018 (30 in 2019 versus 13 in 2018). Since most of the works were based on image processing, CNNs and their variants were chosen in most cases (92.96% among the surveyed literature). From our reviewed work (Tables 2–6), 56 out of the 71 papers (78.87%) performed valid, correct and direct comparisons between the proposed approach and other state-of-the-art techniques with respect to the same problem. The results of these studies indicate that deep learning has better performance in many aspects compared to traditional machine learning for many agricultural tasks^23^.

In terms of evaluating model performance, various performance metrics were employed by the original author(s), and each metric was associated with the model used in each study. At the end of Section “Brief overview of deep learning”, some performance metrics, their definitions/descriptions, and the abbreviations we used in this paper are described. For some studies, where the author(s) directly used accuracy/correct recognition rate without providing its definition, we assumed that they referred to CA. From Tables 2–6, we can see that CA was the most popular metric used (43 papers, 60.56%), followed by F1 (18 papers, 25.35%). Some papers included the RMSE (8 papers), *R*^2^ (3 papers) or other metrics. Twenty-seven of the 71 studies (38.03%) used a combination of performance metrics to evaluate their results. Usually, in combination with CA, F1, precision, recall, or IoU was also used to evaluate the prediction performance of the models.

As each paper adopted different datasets, performance metrics, preprocessing techniques, models and parameters, it is difficult to compare and generalize the results from different papers. Therefore, our comparisons and generalizations would generally be strictly limited to the results from a single paper. However, in this section, we still tried to make some evaluations and comparisons among different technologies, but the readers should take our comments with caution, as the datasets and other parameters used by different authors might not be the same. In 43 of the 71 papers that used CA as a metric, most of the accuracy was higher than 90%, indicating good performance. The highest CA results had values higher than 98%, constituting remarkable results, which were obtained by Amara et al.^41^ (99.72% with a LeNet model), Feng et al.^87^ (99.62-100% with a CNN), Zeng et al.^36^ (99.25% with a modified LeNet model), Brahimi et al.^46^ (99.18% with GoogLeNet and 98.66% with AlexNet), Alruwaili et al.^98^ (99.11% with a modified AlexNet model), Neupane et al.^78^ (98.7% with the Faster R-CNN Inception-V2 model), Giefer et al.^56^ (98.36% with the VGG-16 model), Karthik et al.^92^ (98% with a residual CNN), Bauer et al.^105^ (higher than 98% with a CNN), and Sun et al.^100^ (93.3%–100% with a DBN). Of the surveyed papers, the results obtained by Zhang et al.^85^ had the lowest CA (77.8%–84.5% with an FCN model); however, the SVM model used in this particular task (the detection of internal bruises in blueberries) also obtained a low CA (22.5%–67.9%). Additionally, in Zhu et al.^30^, the AlexNet model (92.1%) obtained CA results that were 14.1% and 11.6% better than the results of the BP neural network (78%) and SVM (80.5%), showing a significant improvement. On the other hand, among the 43 papers that used CA as the metric, AlexNet was the most adopted model (10 papers, 23.2%), followed by VGGNet (8 papers, 18.6%) and ResNet (5 papers, 11.6%). From the 18 papers that used F1 as a metric, 6 papers obtained values above 0.90 and the highest values, which were observed by Ampatzidis et al.^72^ (0.998 with the YOLO-V3 model), Amara et al.^41^ (0.9971 with the LeNet model), and Alruwaili et al.^98^ (0.9929 with a modified AlexNet model), were above 0.99, indicating excellent performance. The results obtained by Wang et al.^48^ had the lowest F1 score (0.531 with an FCF model), but the average running time in the GPU was greatly reduced and was 0.075 s per image.

### Future perspectives of deep learning in the horticulture domain

With the progress of scientific research, deep-learning methods and applications will have a great impact on the horticultural industry and the potential to overcome various challenges (such as productivity challenges, environmental changes, food security and sustainability) in the agricultural industry^5,16,108^. Tables 2–6, which lists a variety of existing applications of deep-learning methods in horticultural science, show that attempts to classify species, detect quality, predict yield and manage pests have been implemented. For example, precise fruit/flower/vegetable detection with deep-learning technology allows the generation of yield maps to provide real-time information on spatial variation from which agronomic decisions can be based to form efficient and precise harvesting strategies for increasing marketable yield^2,105^. This technology would take the place of farmers or gardeners and could solve the current problem of relying heavily on personal experience, which is time consuming, inaccurate and unreliable. Another promising example is the early detection of plant stress^6,90^. Combining deep learning with digital image data or spectral data has shown great potential in improving the speed, accuracy, reliability and scalability of early detection, classification and quantification of plant stress and/or disease^6,16^. In addition, because of the availability of inexpensive digital imaging devices, IoT capabilities, and computing and data storage capabilities, more varieties of horticultural crop information can be used to train deep-learning models and to address some valuable specific issues in the horticultural field.

Although deep learning has superior performance in most of the studies, it is not easy for a reader to quickly choose the right model for a specific task. This difficulty comes not only from the selection of a deep-learning model but also from the hardware and software conditions, weight initialization, learning algorithms, learning rates, activation functions, hyperparameters, validation processes, data sources and data preprocessing methods. Therefore, deep learning can be considered more of an art that relies heavily on personal experience than science. If an established model is to be widely applied to ordinary farms and orchards, it would be necessary to accept that the detection accuracy may be reduced. Since most of the studies used deep learning for image object detection, a CNN was chosen to be successfully applied for the recognition and yield estimation of horticultural plants/products.

There are three main types of CNN-based object detection methods. The first includes some main CNN structures for object recognition, such as LeNet^36,41^, AlexNet^30,62,63^, VGGNet^43,91^, GoogLeNet^46^, and ResNet^31,48^. On the basis of the first type, the second type of method realizes two-stage deep-learning object detection combined with the region proposal method to achieve an improvement in the detection rate and an acceleration in the detection speed; this method type mainly includes the R-CNN^33,73^, Faster R-CNN^44,59,60,77^ and Mask R-CNN^38,54^. The third type includes end-to-end, single-stage deep-learning object detection algorithms, which can directly return the categories and position borders of multiple objects, such as the YOLO^37,72,74^ and SSD^56^ methods. Based on these models, fruit yield can be automatically estimated^32,54,68^, flower and fruitlet thinning and other gardening operations can be automatically conducted^48,101^, and the early detection of plant stress can be accomplished^6,90^.

However, for the quality detection of horticultural products and stress detection of horticultural plants, such as some invisible quality indicators or early bruising detection, a visual analysis method is less effective. From Tables 4 and 5, the authors not only adopted regular CNN methods but also employed other networks, such as the SAE-FNN^27^, CNN-SSAE^86^, DBN^100^ methods, to achieve accurate and rapid feature extraction from a large number of hyperspectral image data and to detect fruit quality (such as SSC and hardness) and plant stress (such as early bruising detection and disease identification). In addition, as Vis/NIR hyperspectral imaging may allow for early detection of plant stress/internal bruising of fruit before symptoms are visible to human eyes, deep-learning approaches would also be promising in this domain. Therefore, deep-learning technology should be combined with these rapid nondestructive testing technologies to explore its great potential in effective feature extraction for direct defect detection. In addition, RNN or LSTM models combined with high-performance regression algorithms or classifiers are promising options that can be used in future horticultural research, especially in yield and price predictions of horticultural products. One example was given in this review that applied LSTM to forecast a banana harvest yield for effective and efficient decision making and forward planning^32^.

Moreover, owing to the advancement of hardware computing capabilities and hardware supply, training processes can be accomplished in much shorter amounts of time. We believe that with the commercialization and widespread use of the Qualcomm Neural Processing SDK (Software Development Kit) for AI and other mobile platforms, handheld smart devices and mobile deep-learning applications will be available for ordinary farms and orchards in the near future. Some of the successful cases deploying deep-learning concepts for plant science applications, such as image recognition and the quality evaluation of horticultural products, early detection systems for plant stress, and yield prediction, can be further transformed for practical application in horticulture^5,16,23^. According to the surveyed papers, some authors^20,58,109^ further integrated tree trunk, branch or fruit recognition algorithms into mobile devices after the model was trained. However, to truly achieve deep learning on mobile devices, some difficulties and problems need to be addressed, such as how to embed a model into mobile devices and how to realize the miniaturization of sensing equipment. Thus, more research is needed to achieve this goal.

Currently, deep-learning technology has been able to open the door to intelligent horticulture for difficult gardening jobs. Based on these precise phenotypic data, we can accurately monitor crop growth features at different critical stages and key yield-related traits and implement precise agricultural decision management. As shown in Table 1, the performance of deep learning combined with image data has steadily improved, and these models have also been successfully applied in horticultural science. However, as was described before, it is a difficult task to choose the most suitable model among the various techniques that have been proposed to date for a specific application in horticultural science. In addition, there is also an urgent need to build large datasets containing plant images to create robust models. When collecting plant phenotypic data, we strongly recommend the use of in-field real condition imaging data (i.e., with varying shade, light, and mutual occlusion conditions) to create training datasets. Correct labeling and the open-source use of these datasets can avoid the duplication of data collection. With the increasing amount of collaborative research and the joint effort among horticulturalists and computer scientists, we are confident that deep-learning technology has great potential to support the horticulture industry more intelligently and accurately to improve yield and quality and to better detect plant stress and diseases.

## Conclusion

With the rapid explosion of data in horticultural sciences, deep-learning technology has become a hot research focus and has opened a new area in artificial intelligence. Deep-learning methods provide a powerful tool to assimilate data and have proven to hold promise for overcoming the existing challenges to record plant growth objectively, judge plant status accurately and detect the quality of products quickly in horticultural science. A key element for the successful large-scale application of deep-learning technology lies in the joint effort of scientists from both computer and horticulture sectors and the seamless integration of data collection along with an effective curation pipeline^14^. Such efforts would allow for the formation of a computational ecosystem that might provide tremendous opportunities to facilitate planting, promote intelligent orchard management and tackle other problems. Some of the solutions discussed in this paper also have potential for commercialization in the near future. For example, automatic robots incorporated with a faster region-based CNN could be used in transplanting, fruit picking or yield estimation. The aim of this review is to introduce this relatively new and effective tool so that researchers and workers in horticulture sectors can manage the massive amounts of data they might collect in their research and to encourage researchers to use or improve data to solve their problems to gradually to move towards a smart horticulture industry.
